# Identification and profiling of upland cotton microRNAs at fiber initiation stage under exogenous IAA application

**DOI:** 10.1186/s12864-019-5760-8

**Published:** 2019-05-28

**Authors:** Tianlun Zhao, Xiaojian Xu, Min Wang, Cheng Li, Cong Li, Rubing Zhao, Shuijin Zhu, Qiuling He, Jinhong Chen

**Affiliations:** 1 Department of Agronomy, Zhejiang University, Zhejiang, 310058 Hangzhou China; 2 Zhejiang Province Key Laboratory of Plant Secondary Metabolism and Regulation, Zhejiang Sci-Tech University, Zhejiang, 310018 Hangzhou China

**Keywords:** Upland cotton (*Gossypium hirsutum* L.), MicroRNA, Fiber initiation, Exogenous IAA

## Abstract

**Background:**

Cotton is the most essential textile crop worldwide, and phytohormones are critical for cotton fiber development. One example is the role of auxin in fiber initiation, but we know little molecular basis. MicroRNAs (miRNAs) have a significant function in cotton development; nevertheless their role in fiber initiation remains unclear. Here, exogenous IAA was applied to cotton plant before anthesis. Utilizing small RNA sequencing, the mechanism underlying miRNA-mediated regulation of fiber initiation under exogenous IAA treatment was investigated.

**Results:**

With exogenous IAA application, the endogenous IAA and GA contents of IAA treated (IT) ovules were higher than control (CK) ovules at the fiber initiation stage, while endogenous ABA content was lower in IT than CK. Using scanning electron microscopy, we found the fiber number and size were significantly promoted in IT at 0 DPA. Fiber quality analysis showed that fiber length, uniformity, strength, elongation, and micronaire of IT were higher than CK, though not statistically significant, while lint percent was significantly higher in IT. We generated six small RNA libraries using − 3, 0, and 3 DPA ovules of IT and CK, and identified 58 known miRNAs and 83 novel miRNAs together with the target genes. The differential expressed miRNAs number between IT and CK at − 3, 0, 3 DPA was 34, 16 and 24, respectively. Gene ontology and KEGG pathway enrichment analyses for the target genes of the miRNAs expressed in a differential manner showed that they were significantly enriched in 30 terms and 8 pathways. QRT-PCR for those identified miRNAs and the target genes related to phytohormones and fiber development was performed, and results suggested a potential role of these miRNAs in fiber initiation.

**Conclusions:**

The exogenous IAA application affected the relative phytohormone contents in ovule and promoted fiber initiation in cotton. Identification and profiling of miRNAs and their targets at the fiber initiation stage provided insights for miRNAs’ regulation function of fiber initiation. These findings not only shed light on the regulatory network of fiber growth but also offer clues for cotton fiber amelioration strategies in cotton.

**Electronic supplementary material:**

The online version of this article (10.1186/s12864-019-5760-8) contains supplementary material, which is available to authorized users.

## Background

Cotton (*Gossypium hirsutum* L.) has been listed among the foremost economic crops in the world, which offers the greatest quantity of renewable resources for the cottonoracy. Cotton fibers refer to extremely elongated single-cell trichomes initiated from ovule epidermis. Development of cotton fiber has four differing stages with overlapps: initiation, elongation, secondary wall thickening and maturation [1, 2]. Typically, fiber initiation begins at the day of anthesis (0 DPA) and sustains for ~ 5 days. The number of fiber initials determines the number of fibers, and the initiation stage is thus the determining stage contributing to the final yield of cotton [3]. Fibers start elongation immediately after initiation and continue for ~ 25 days. Fibers enter the secondary wall thickening stage at about 20 DPA, and maturation stage at 35 DPA, respectively [4–6].

Phytohormones are quite essential for fiber development in cotton. It has been shown that gibberellins (GAs) not only have a positive effect on the fiber initiation and elongation, but also contribute to fiber secondary wall deposition [7, 8]. Ethylene facilitates fiber elongation, probably because of the enhance expression level of key genes in relation with sucrose synthase, tubulin, and expansin. In contrast, fiber growth was found to be inhibited by abscisic acid (ABA) in cotton ovule culture [9]. Indole-3-acetic acid (IAA), is a widespread natural auxin, that is critical for fiber development [10, 11]. Previous studies reported that auxin could promote fiber initiation and fiber units when applied to ovules exogenously [11, 12]. Pioneering in vivo experiments revealed that overexpression of a gene responsible for the auxin biosynthesis could simultaneously improve the yield and quality of cotton fiber [13]. In an previous experiment, with exogenous IAA application under field conditions, fiber number per ovule significantly increased [14]. Despite these findings, the complex mechanism by which auxin regulates fiber development requires further study.

MicroRNAs (miRNAs) are short noncoding RNAs with length ~ 22 nt. They negatively regulate the expression or translation of their target genes [15, 16]. MiRNAs are usually derived from intergenic regions as well as introns of genomes, and they are initially transcribed into primary miRNAs (pri-miRNAs) mainly by RNA polymerase II [17]. Pri-miRNAs are successively processed twice by a protein complex, including Dicer-like 1 (DCL1), into pre-miRNAs (miRNA precursors) with a typical hairpin structure, and then into mature miRNAs [18]. Mature miRNAs are loaded onto RNA-induced silencing complex (RISC) including AGO1 protein, and complementary binding to their target mRNAs for cleavage or translation inhibition [19, 20]. In the RNA-induced silencing process, miRNA has a guidance role through base pairing with the target mRNAs, while AGO proteins undertake an effectors role through recruitment of factors inducing translational repression, mRNA deadenylation and mRNA decay [21]. Extensive research have proved the miRNAs have a basic regulatory function of miRNAs in diver biological processes, including plant organ development, hormone signal transduction, stress tolerance and disease resistance [20, 22–27].

Recent studies of small RNAs in cotton have identified and characterized numerous miRNAs. In a study investigating the regulation of cotton plant height, An et al. sequenced small RNAs, identified 226 conserved miRNAs belonged to 32 known miRNA families along with 38 novel miRNAs, and found that several miRNAs were regulated in different height-related mutants [28]. As for cotton fiber, Pang et al. sequenced small RNAs from cotton fiber and other tissues and made identification of 27 conserved, four novel miRNA families [29]. In an investigation of fiber elongation, by sequencing the developing fiber of short fiber mutants *Li*_*1*_ and *Li*_*2*_, 24 conserved and 147 novel miRNA families along their targets were revealed_,_ and four miRNAs were found to be negatively correlated with fiber lengths [30]. In *Gossypium arboreum*, Liu et al. found 48 known and 16 novel miRNAs, which expressed in elongating fibers in a differential manner [31]. Using a similar small RNA sequencing strategy, the present study focuses on the key role of exogenous IAA in fiber initiation, and miRNA-mediated regulation of fiber initiation. To date, there have not been any systematic studies using miRNA expression profiling and target genes analysis upon application of exogenous IAA during fiber initiation.

Here, we sequenced six libraries from − 3, 0, and 3 DPA ovules of control (CK) and IAA treated (IT) cotton at fiber initiation stage, and bioinformatics analyses were carried out for discovering of known and novel miRNAs. Based upon the expression profiles of the selected miRNAs together with the relevant target genes, we showed potential roles of some miRNAs in fiber initiation, and provide data for a deeper understanding of the molecular mechanism of miRNA in fiber initiation.

## Methods

### Plant materials

The genetic-standard upland cotton TM-1 was kindly provided by USDA-ARS, College Station, Texas, USA. This material was grown at the farm of Zhejiang University, China. At the day of the day of anthesis (0 DPA), flowers were labeled. The concentration of exogenous IAA was 5*10^− 6^ mol/L, and then this IAA solution was sprayed onto the cotton plant for three times every other day. After 3 days, bolls at − 3, − 1, 0, 1, 2, 3 and 5 DPA were harvested, and each of them were separated into three biological repeats randomly. Plants without exogenous IAA treatment were used as control group. After careful dissection from every boll, the ovules were frozen in liquid nitrogen immediately and stored at − 80 °C. The following six samples were used for the small RNA sequencing. C_1, C_2 and C_3 were the ovules of − 3, 0 and 3 DPA TM-1 without exogenous IAA. I_1, I_2 and I_3 were the ovules of − 3, 0, 3 DPA TM-1 with exogenous IAA treatment.

### Phytohormone determination

Contents of GA, IAA, and ABA were measured using high performance liquid chromatography (HPLC). Standard samples were dissolved in the mobile phase containing methanol/ 0.2% phosphoric acid (1/1, v/v) to a constant volume of 1 ml.

All samples were weighed (1 g) and grounded into powder with liquid nitrogen. Samples were suspended into 4 ml acetonitrile and leached at 4 °C for 24 h. The supernatant was obtained by centrifugation with the speed of 1000 r/m for 10 min, and the sediment was washed thrice with acetonitrile. All the supernatants should be combined and dried by nitrogen at 35 °C. 3 ml volume of phosphoric acid buffer was added, the solution was centrifuged at 1000 r/m for 15 min, and the pH of the filtrate was adjusted to 3. An equal volume of ethyl acetate was used for extraction twice. The obtained organic phase was dried by nitrogen at 35 °C and added to the mobile phase to constant volume of 1 ml. 0.45 syringe filter (Agela, Newark, USA) was used for solution filtering.

Agilent was used for HPLC analysis of phytohormone, and the method was as previous reported by Cheng et al. [32]. Samples were measured in triplicates.

### Small RNA libraries construction and sequencing

Extraction of total RNA was conducted out of ovules by utilizing a Total RNA Extraction kit (Aidlab, Beijing, China) in line with the manufacturer’s protocol. 1% agarose gels was performed for inspection of the RNA contamination and degradation. NanoPhotometer® spectrophotometer (IMPLEN, CA, USA) was used to examine the RNA purity. Qubit® RNA Assay Kit on Qubit® 2.0 Fluorometer (Life Technologies, CA, USA) was utilized for detecting the RNA concentration. Nano 6000 Assay Kit of the Agilent Bioanalyzer 2100 system (Agilent Technologies, CA, USA) to test the RNA integrity.

For every sample, 3 μg total RNA was applied for the construction of Small RNA library. With the manufacturer’s instruction, the NEBNext® Multiplex Small RNA Library Prep Set for Illumina® (NEB, USA.) was employed for generating of the sequencing library. Lastly, Illumina HiSeq 2500/2000 platform was used to sequence the library, thereby generating 50 bp single-end reads.

### Analysis of small RNA sequencing data

Fastq format raw reads had been initially processed with application of custom Perl and Python scripts. During such procedure, clean reads would be acquired through removal of the following reads: reads containing poly-N, with 5′ adapter contaminants, without 3′ adapter or the insert tag; reads containing poly A, T, G, or C; and low quality reads from raw data. Meanwhile, calculation of Q20, Q30, and GC-content of the raw data was performed. Clean reads 18 to 30 nt in length were selected. For removal of tags that originated out of protein-coding genes, repeat sequences, rRNA, tRNA, snRNA, and snoRNA, the small RNA tags were mapped to RepeatMasker, Rfam database, or such kinds of data from the certain species itself. The small RNA tags were mapped onto the reference sequence using Bowtie [33] without mismatches for analysis expression and distribution on the reference sequence.

### Identification of known miRNAs and prediction of novel miRNAs

To search the known miRNAs among the mapped small RNA sequences, miRBase21.0 was applied as the reference, utilization of mirdeep2 [34] and srna-tools-cli software was performed for making a prediction of the secondary structure of the flanking sequences of small RNAs matching known miRNAs. The first-position base of the identified miRNAs with specific length, bases upon every position of all identified miRNAs, and miRNA counts were calculated by custom scripts. Since novel miRNAs were predictable by the hairpin structure of miRNA precursors, miREvo [35] and mirdeep2 software were utilized together for novel miRNAs prediction based on the dicer cleavage site, the secondary structure, and the minimum free energy of the small RNA sequences, which was unannotated in the known miRNAs identification steps.

### Prediction of miRNA target genes

MiRNA target gene prediction was carried out by psRobot_tar in psRobot [36].

### Analysis of differentially expressed miRNAs

TPM was utilized for estimating the miRNA expression level using the formula herein under [37]: TPM = (mapped readcount/total reads)*1,000,000. Differential expression (log2 (fold-change) > 1) of miRNAs in the two samples should be assessed by DEGseq (2010) R package. Adjustment of *P*-value should be done with application of qvalue (< 0.01) [38].

### GO and KEGG pathway enrichment analyses

Gene ontology (GO) enrichment analysis was employed for surveying the target genes of miRNAs expressed in a differential manner. GOseq, which was based Wallenius non-central hyper-geometric distribution [39], was carried out to perform a GO enrichment analysis.

KEGG [40] serves as a biological database, which provides the information of genomic, chemical and systemic (http://www.genome.jp/kegg/). It is for investigating biological system functions and utilities [41]. KOBAS software would be employed for testing for statistically significant enrichment of the target genes among the KEGG pathways.

### Expression analysis of selected miRNAs and their target genes using real-time qPCR

Extraction of RNA samples was performed by Total RNA Extraction kit (Aidlab, Beijing, China). The reaction of reverse transcription for miRNAs was carried out with Mir-XTM miRNA First-Strand Synthesis kit (TaKaRa, Dalian, China). First strand cDNA synthesis of target genes should be conducted through utilization of PrimeScript™ First-Strand cDNA Synthesis Kit (TaKaRa, Dalian, China). Design of primers was made for qRT-PCR through Primer 5.0 software. All primers have been listed in Additional file 1: Table S1. The qRT-PCR reactions of miRNAs were carried out on the Lightcycler 96 system (Roche) with TB Green *Premix* Ex *Taq* (TaKaRa). Each reaction was carried out in triplicate. *U6 and UBQ7* were employed to be internal control miRNA and internal control gene, respectively. Calculation of the relative expression level was conducted with utilization of the 2^-ΔΔCt^ method.

## Results

### Effect of exogenous IAA on the content of endogenous phytohormones

**Fig. 1:**
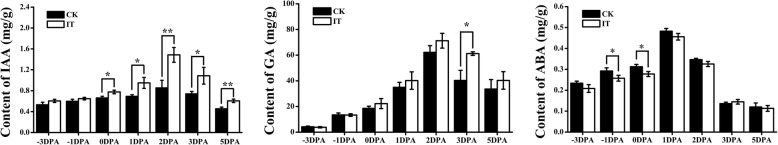
The contents of endogenous phytohormones including IAA, GA and ABA in control (CK) and IAA treated (IT) ovules. * indicates *P* < 0.05

To investigate the effect of exogenous IAA on cotton fiber and phytohormones, we sprayed IAA on cotton plants at − 9, − 7, and − 5 DPA. The analysis revealed significant differences in the endogenous phytohormone contents between CK and IT ovules (Fig. 1). At − 3 and − 1 DPA, there were no significant differences in endogenous IAA content of CK and IT. However, the endogenous IAA contents of IT significantly greater than that of CK at 0, 1, and 3 DPA, along with markedly significant difference at 2 and 5 DPA, indicating that exogenous IAA promoted the secretion of endogenous IAA. For endogenous GA content, the exogenous IAA application generally promoted the endogenous GA content at the fiber initiation stage. The endogenous GA content in IT was significantly greater than in CK at 3 DPA, while the contents in IT were higher than in CK at 0, 1, 2, and 5 DPA, but not significantly. It was suggested that exogenous IAA might have a positive impact on endogenous GA. For endogenous ABA content, it is generally depressed by the exogenous IAA application. The endogenous ABA contents in IT was significantly lower than in CK at − 1 and 0 DPA, but there were no significant differences between IT and CK at other time point. Thus, exogenous IAA might have a negative impact on endogenous ABA at the early stage of initiation.

### Effect of exogenous IAA on fiber initiation and fiber quality

**Fig. 2:**
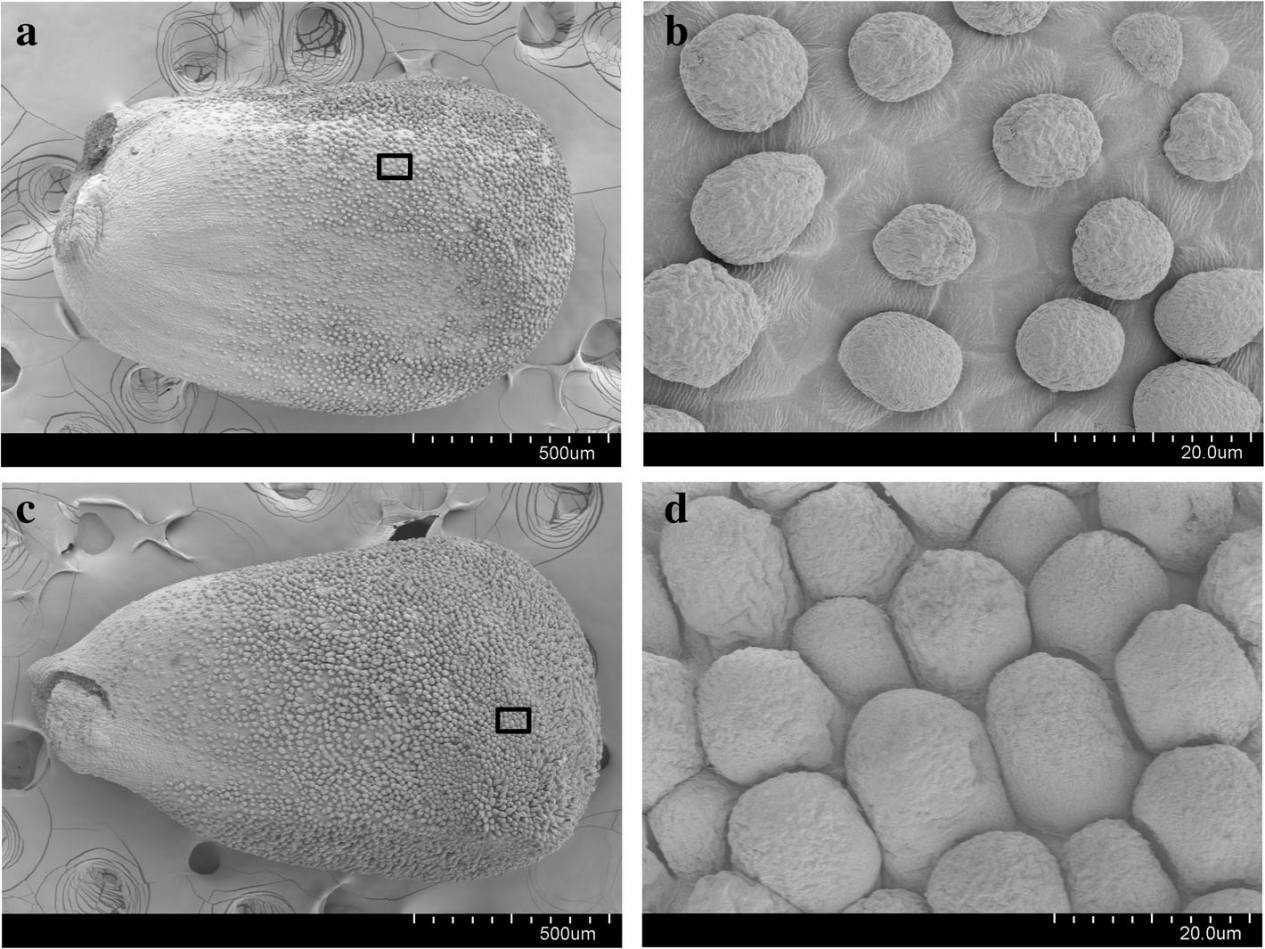
Differences in the initial development of fibers at 0 DPA between control (CK) and IAA treated (IT) plant. **a** and **b** show CK ovules surfaces at 80× and 2000× magnification; (**c)** and (**d**) show IT ovules surfaces at 80× and 2000× magnification. **b** and **d** are the black frame aeras of (**a)** and C. It was indicated that the number of lint fiber on ovules of IT was much higher than that of CK, and the size of of lint fiber on ovules of IT was much larger than that of CK

To further investigate the impact of exogenous IAA on the cotton fiber, we observed the development of fiber cells by scanning electron microscopy (SEM) of ovule surface at 0 DPA. As shown in Fig. 2, the number of fiber initials on IT ovules was significantly greater than on CK ovule. According to the statistical result, lint fiber number on CK and IT ovules were 4733 and 3867 per mm^2^, respectively, and lint fiber number on IAA ovules was significantly higher than that on IT ovules. Moreover, the sizes of lint fibers on IT ovules were also significantly larger than those on CK ovules. Above all, it is suggested that exogenous IAA facilitated fiber initiation and early development at the fiber initiation stage.

Mature fiber quality traits including fiber length (FL), fiber uniformity (FU),, elongation (FE), strength (FS) and micronaire (MIC) were also measured. As shown in Table 1, FL, FU, FE and FS values tended to be higher in IT compared to CK, but the differences were not significant. MIC and lint percent (LP) in CK were 3.63 and 32.41%, respectively, while MIC and LP in IT were 3.90 and 34.07%, which was significantly higher than those in CK. These results indicated that exogenous IAA had an impact on mature fiber quality and positive effect on lint percent.

**Table 1:** Mature fiber quality trait in control (CK) and IAA treated (IT) plants (LOG 298 bytes)

	FL (%)^a^	FU (%)^a^	FS (cN/tex)^a^	FE (%)^a^	MIC^a^	LP (%)^a^
CK	26.47 ± 0.51	83.93 ± 1.24	23.84 ± 0.44	6.43 ± 0.06	3.63 ± 0.06	32.41 ± 0.76
IT	26.73 ± 0.12	84.20 ± 0.61	24.23 ± 0.32	6.43 ± 0.06	3.90 ± 0.10*	34.07 ± 0.57*

*Significant at *P* = 0.05

^a^*FL* fiber length, *FU* fiber uniformity, *FS* fiber strength, *FE* fiber elongation, *MIC* micronaire, *LP* lint percentage

### Deep sequencing of small RNA libraries from developing ovules

**Fig. 3:**
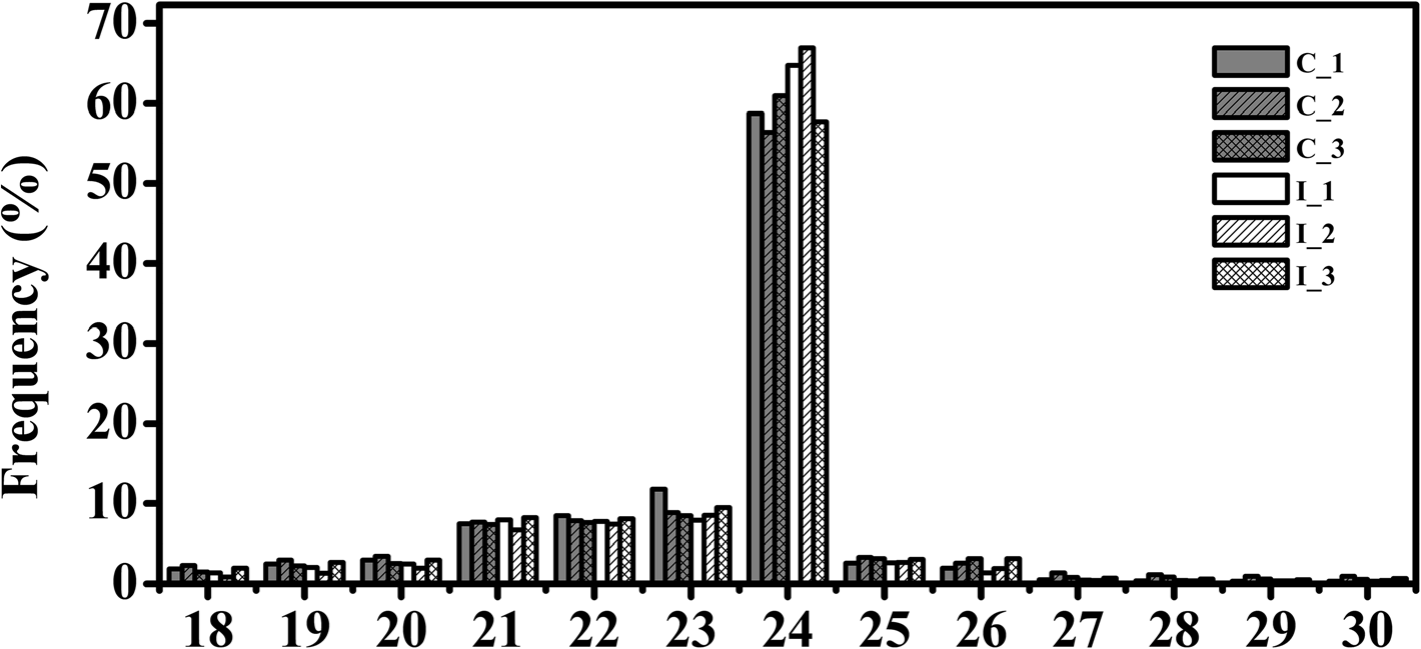
Sequence length distribution of small RNAs from six libraries. Libraries of C_1, C_2, C_3, I_1, I_2, and I_3 were constructed from control and IAA treated ovules at − 3, 0, and 3 DPA. The 24 nt small RNA group is much larger than the others

To identify miRNAs involved in cotton fiber initiation under exogenous IAA treatment, conduction of six small RNA libraries was performed out of IT and CK ovules of − 3, 0 and 3 DPA. A total of 70.98 million raw reads were generated, and after quality control and length selection of the reads, 9,655,792, 10,836,541, 10,112,797, 11,174,398, 10,823,945 and 9,611,924 small RNAs were obtained from C_1, C_2, C_3, I_1, I_2 and I_3, respectively (Table 2). On average, 76.75% of the reads were successfully aligned to the AD genome of *G. hirsutum* following the removal poor quality reads and adapter sequences. Furthermore, the sRNAs were categorized to be a couple of major classes. Those unique reads were very similar among the six libraries and ranged from 18 to 30 nt after the filtering process, the length of most sRNAs was 20-24 nt, with the 24 nt reads being the most abundant (Fig. 3).

**Table 2:** Classification statistics of small RNA sequence reads in six small RNA libraries

Types	C_1	C_1(percent)	C_2	C_2(percent)	C_3	C_3(percent)	I_1	I_1(percent)	I_2	I_2(percent)	I_3	I_3(percent)
Total sRNA	9,655,792	100.00%	10,836,541	100.00%	10,112,797	100.00%	11,174,398	100.00%	10,823,945	100.00%	9,611,924	100.00%
Mapped sRNA^a^	7,698,290	79.73%	8,215,859	75.82%	7,777,656	76.91%	8,167,793	73.09%	7,860,750	72.62%	7,910,588	82.30%
known_miRNA	51,514	0.53%	49,264	0.45%	53,190	0.53%	74,506	0.67%	54,174	0.50%	58,854	0.61%
rRNA	110,620	1.15%	299,739	2.77%	157,689	1.56%	103,787	0.93%	63,869	0.59%	163,763	1.70%
tRNA	0	0.00%	0	0.00%	0	0.00%	1	0.00%	0	0.00%	0	0.00%
snRNA	6671	0.07%	9880	0.09%	21,580	0.21%	9224	0.08%	8331	0.08%	7597	0.08%
snoRNA	5204	0.05%	8557	0.08%	8502	0.08%	7175	0.06%	5541	0.05%	6516	0.07%
repeat	1,396,437	14.46%	1,960,782	18.09%	1,438,013	14.22%	1,395,089	12.48%	1,227,994	11.35%	1,462,223	15.21%
NAT	2506	0.03%	4680	0.04%	3696	0.04%	2736	0.02%	2926	0.03%	4428	0.05%
novel_miRNA	68,759	0.71%	56,358	0.52%	52,532	0.52%	104,195	0.93%	62,573	0.58%	53,194	0.55%
TAS	1118	0.01%	1416	0.01%	1986	0.02%	1367	0.01%	1382	0.01%	1646	0.02%
exon:+	142,196	1.47%	160,388	1.48%	176,661	1.75%	181,023	1.62%	157,999	1.46%	175,040	1.82%
exon:-	81,025	0.84%	72,668	0.67%	75,584	0.75%	87,726	0.79%	79,701	0.74%	75,401	0.78%
intron:+	521,977	5.41%	479,031	4.42%	466,814	4.62%	550,317	4.92%	535,491	4.95%	483,474	5.03%
intron:-	361,929	3.75%	374,424	3.46%	382,396	3.78%	426,582	3.82%	393,663	3.64%	406,575	4.23%
other^b^	4,948,334	51.25%	4,738,672	43.73%	4,939,013	48.84%	5,224,065	46.75%	5,267,106	48.66%	5,011,877	52.14%

^a^Contains all the classified sequences, including known miRNA, rRNA, tRNA, snRNA, snoRNA, repeat, NAT, novel_miRNA, TAS, exon:+, exon:-, intron:+, intron- and other

^b^Contains all of the unclassified sRNA reads

### Identification of known miRNAs and prediction of novel miRNAs

For discovering known miRNAs in the six libraries, the mapped small RNA tags were aligned to all mature miRNA and miRNA precursor sequences in miRBase21.0. For CK and IT, 1974 and 2061 specific sequences had been identified as miRNA homologies in CK and IT plants, respectively. Following the removal of repeated sequences, 58 annotated known miRNAs under 50 miRNA families had been identified (Additional file 2: Table S2). Specifically, 53, 53, 52, 51, 52, and 54 of these miRNAs were distributed in C_1, C_2, C_3, I_1, I_2 and I_3, respectively. Three miRNAs, ghr-miR3476-3p, ghr-miR7488, and ghr-miR7497, were found exclusively in IT plants, and ghr-miR7498 was only present in CK plants.

The sequences failing to match known miRNAs were further compared with the cotton genome sequence to identify potential novel miRNA candidates using miREvo and mirdeep2 software [35]. Upon the basic of the characteristic hairpin structure of miRNA precursors, 84 novel miRNAs were identified among all six libraries; the sequences and stem-loop structures are included in Additional file 3: Table S3 and Additional file 4. The lengths of the novel miRNAs ranged from 18 to 24 bp, with 21 nt miRNAs comprising the greatest category, with subsequent by 24 nt miRNAs. First nucleotide base analysis was performed for these novel miRNAs and the results are summarized in Table 3. In the analysis, adenine (A, 36.14%) and uracil (U, 53.01%) were the most dominant nucleotides at the first position.

**Table 3:** The firstbase and lengths of the 83 novel cotton miRNAs

Firstbase	miRNA length (nt)							
	18	19	20	21	22	23	24	Total
A	1	0	0	5	5	0	19	30
U	0	0	4	29	10	0	1	44
C	1	0	1	4	0	0	0	6
G	0	0	0	2	0	0	1	3
Total	2	0	5	40	15	0	21	83

### Targets of known and novel miRNAs

Plant miRNAs bind to their targets with perfect or nearly perfect complementarity to direct post-transcriptional repression via mRNA cleavage or translation repressing. Therefore, miRNAs target genes are predictable upon the basic of the principle of high miRNA-target complementarity. psRobot was used to predict putative target genes, and 911 targets were identified for 109 miRNAs, with 437 target genes for 47 known miRNAs and 614 target genes for 63 novel miRNAs (Additional file 5: Table S4). Most miRNAs processed diverse target genes, such as Gh_A01G1274, Gh_A01G1281, and Gh_A03G0632 targeted by ghr-miR156a. Such miRNAs might therefore play a key role in cotton fiber initiation. From another point of view, there were also examples of a single gene targeted by several miRNAs, such as Gh_A03G0276 targeted by ghr-miR166b, ghr-miR1, and ghr-miR3.

In other cases, miRNAs such as ghr-miR7486a and ghr-miR15 had no predicted targets, suggesting that they might regulate gene expression through distinct mechanisms, such as translation inhibition.

### Expression of known and novel miRNAs

**Fig. 4:**
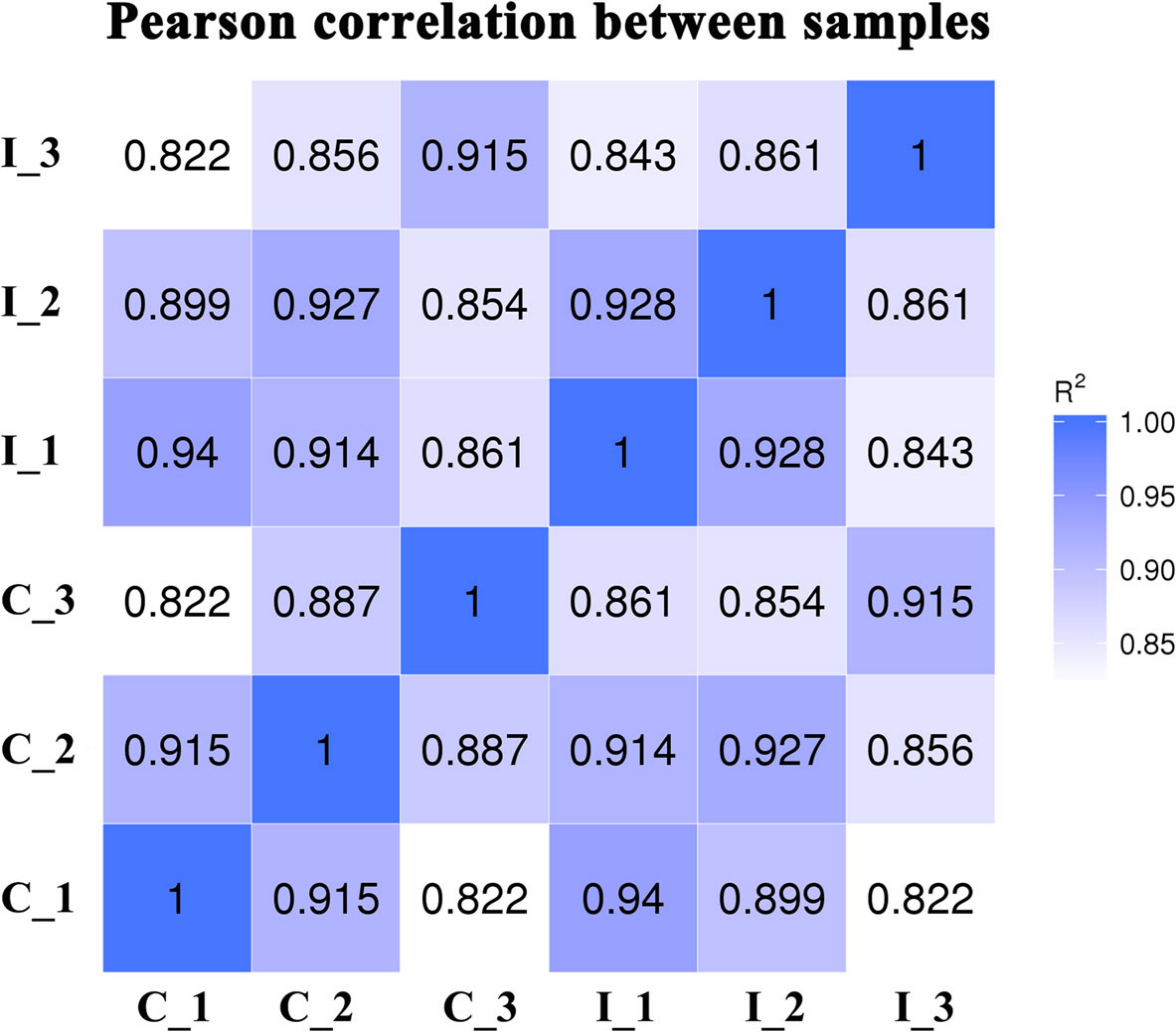
Pearson correlation of miRNA expression levels among six samples. Ovule samples of control and IAA treated at − 3, 0, and 3 DPA were named as C_1, C_2, C_3, I_1, I_2, and I_3. Correlation coefficients closer to a value of 1 indicate greater similarity in the expression patterns of two samples

Correlations among the six libraries were analyzed using Pearson’s correlation, and the results are shown in Fig. 4. The correlation coefficient between adjacent phases was higher than spaced ones both in the CK group (C_1, C_2 and C_3) and IT group (I_1, I_2 and I_3), and within groups as well. On the same day, the correlation coefficient within the same group was higher than that in different group. These findings revealed the dynamic trend of miRNA within the same group and the effect of IAA on miRNA alteration.

**Fig. 5:**
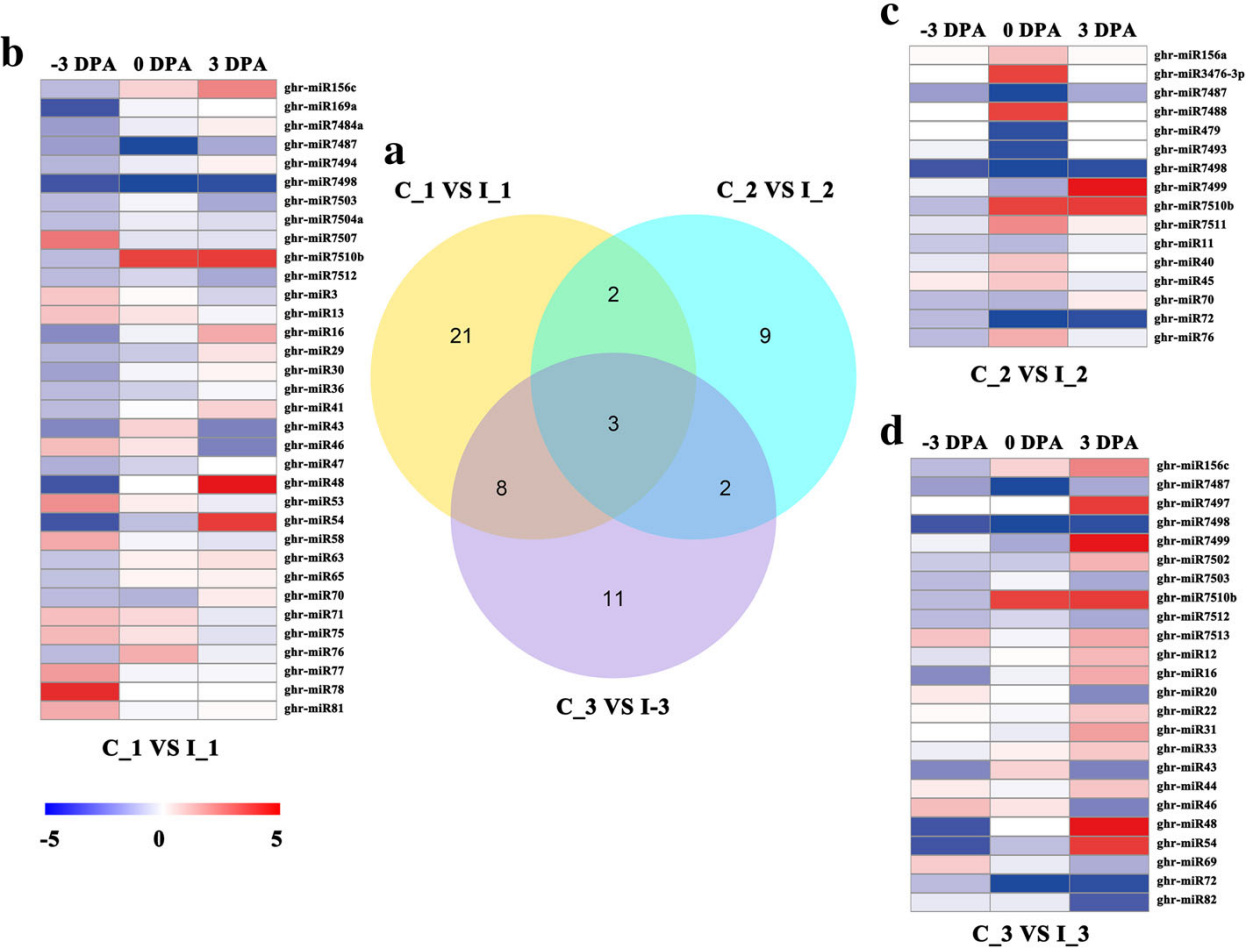
Differential expressed miRNAs at − 3, 0 and 3 DPA. **a** showed the venn diagram of the differential expressed miRNAs between control (CK) and IAA treated (IT) at − 3, 0 and 3 DPA (C_1 VS I_1, C_2 VS I_3 and C_3 VS I_3), respectively. Three circles represented three comparative combinations. Sum of the numbers in the circles was the number of the differential expressed miRNAs in the circles corresponding comparative combinations. Sum of the numbers in the overlapping part of circles was the number of the common differential expressed miRNAs in different combinations. **b**, **c** and **d** showed heatmap of different expressed miRNAs between CK and IT at − 3, 0 and 3 DPA. The color indicates the fold-change as log2, from high (red) to low (blue), as indicated in the color scale. On the right are the names of the miRNAs

For discovering the differential expressed miRNAs between CK and IT, we detected the abundance of miRNA reads, and carried the veen diagram of the number of the differential expressed miRNAs and the heatmaps of the differential expressed miRNAs at − 3, 0 and 3 DPA (Fig. 5). As shown in Fig. 5a, altogether 56 miRNAs were expressed in a differential manner between CK and IT. At − 3 DPA, 34 differentially expressed miRNAs were identified, containing 11 known miRNAs and 23 novel miRNAs, among those 11 miRNAs were up-regulated and 23 miRNAs were down-regulated in IT (Fig. 5b). At 0 DPA, 16 miRNAs expressed in a differential manner, containing 10 known miRNAs and 6 novel miRNAs, and 8 miRNAs were up-regulated and eight miRNAs were down-regulated in IT (Fig. 5c). At 3 DPA, 24 miRNAs expressed in a differential manner, containing 10 known miRNAs and 14 novel miRNAs, and 14 miRNAs were up-regulated and 10 miRNAs were down-regulated in IT (Fig. 5d). Among the differentially expressed miRNAs, 58.11% belong to novel miRNAs, some of which have an essential function in the mechanism of IAA-regulated fiber initiation regulated by IAA.

**Fig. 6:**
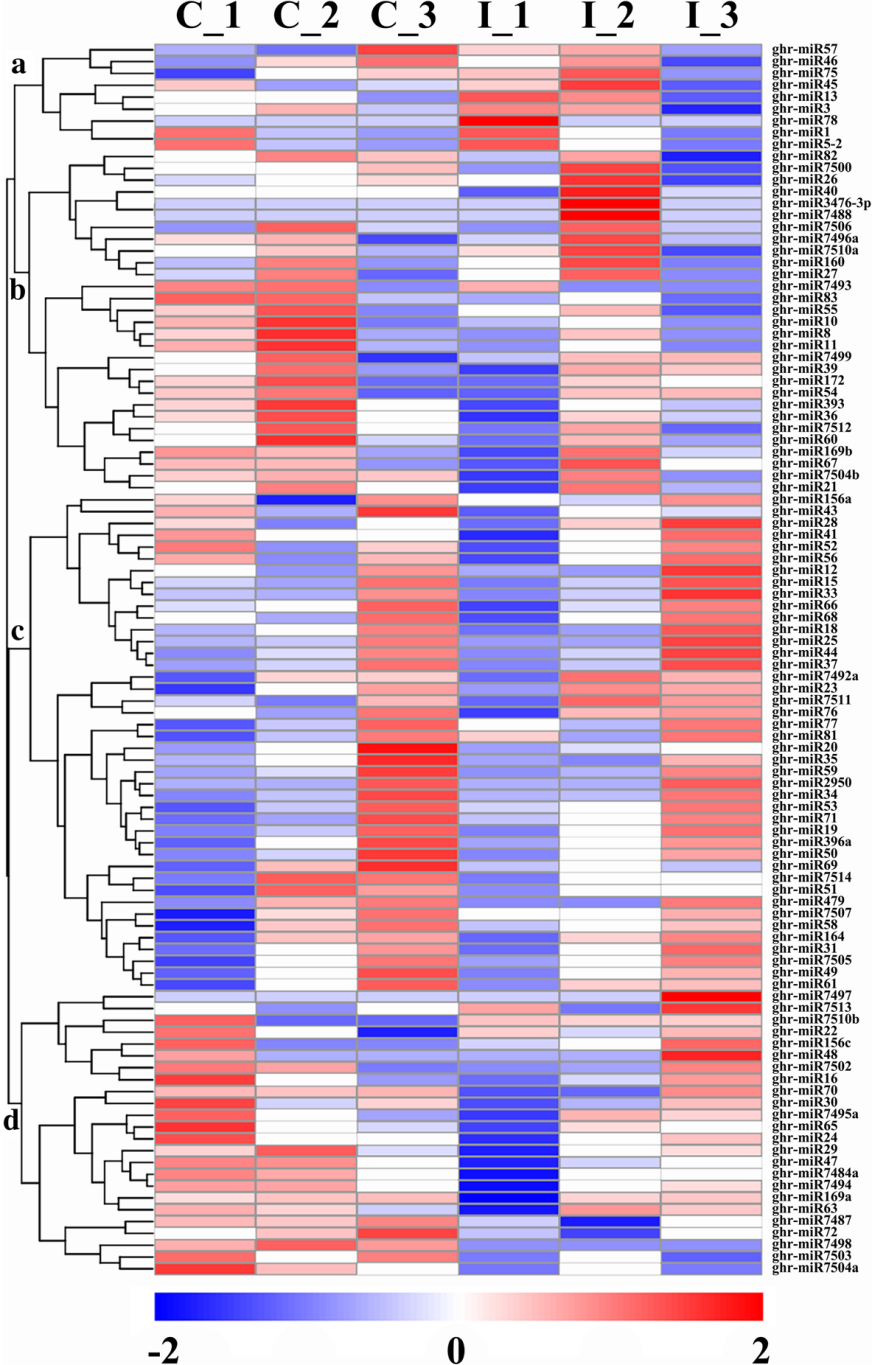
Complete hierarchical cluster analysis of differentially expressed miRNAs in cotton ovules. The analysis was performed by comparing the TPM of miRNAs among control and IAA treated (− 3, 0 and 3 DPA) libraries (C_1, C_2, C_3, I_1, I_2, and I_3). The color indicates the log_10_ (TPM + 1) change from high (red) to low (blue), as indicated in the color scale. MiRNA names are on the right, and the classes (**a**-**d**) they belong are shown on the left

According to the transcript per million (TPM) of differentially expressed miRNAs among six libraries, we merged the differential expressed miRNAs in all compared pairs of CK and IAA at − 3, 0 and 3DPA together to perform cluster analysis. As shown in Fig. 6, the miRNA expression patterns clustered into four classes for both known and novel miRNAs using the hierarchical clustering method. Class A comprised nine miRNAs and the expression of ghr-miR78, ghr-miR1, and ghr-miR5–2 decreased over time both in CK and IT. In class B, 29 miRNAs were clustered. Many miRNAs, including ghr-miR83, ghr-miR55, ghr-miR8, and ghr-miR7512, were down-regulated in IT. In class C, 42 miRNAs were clustered, and the expression levels of many miRNAs increased over time, like ghr-miR31, ghr-miR7505, ghr-miR49, and ghr-miR61. Class D comprised 24 miRNAs. The expression of several miRNAs decreased over time in CK and increased in IT, such as ghr-miR7502 and ghr-miR16. The potential involvement of these miRNAs in fiber initiation will require further research.

### Functional annotation and classification

**Fig. 7:**
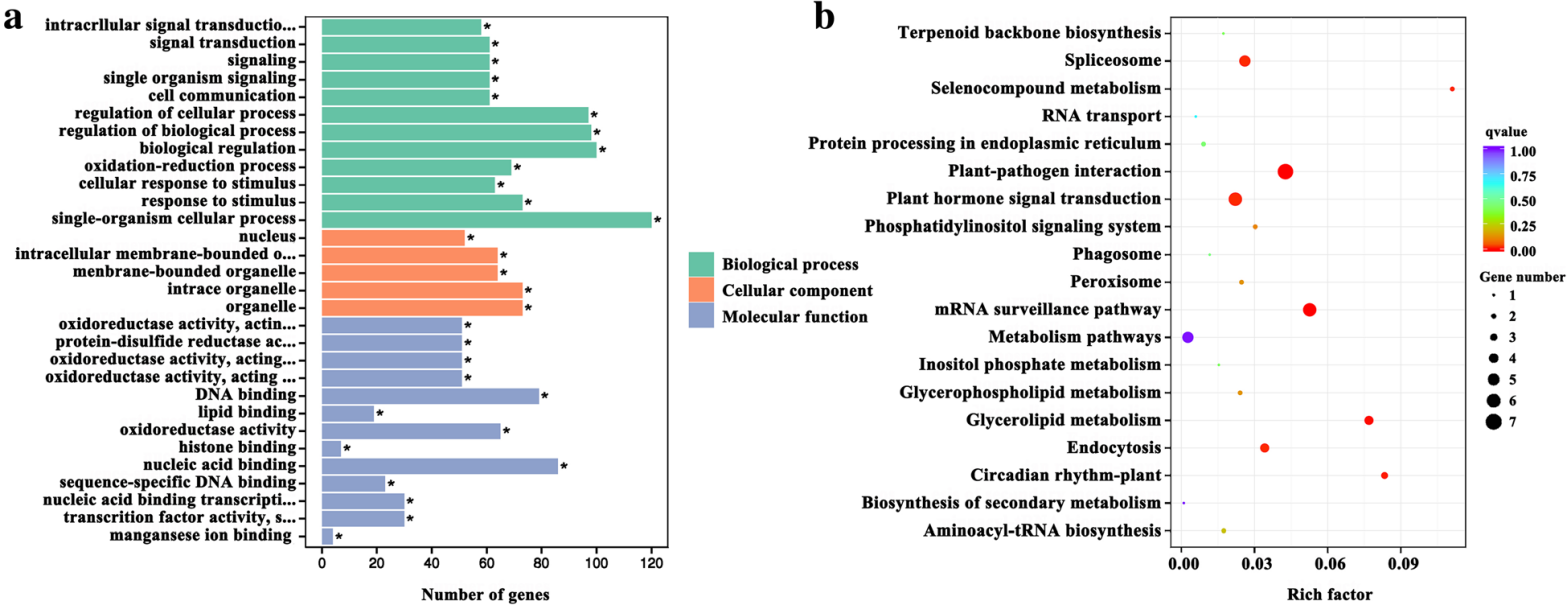
GO and KEGG pathway enrichment analyses of total target genes of differentially expressed miRNAs at − 3, 0 and 3 DPA. **a** presents the GO enrichment analysis result (green: biological process; orange: cellular component; blue: molecular function), and * indicates *P* < 0.05. **b** presents the KEGG pathway analysis result, and the color indicates the qvalue change from 0 to 1 as indicated in the color scale and the size of the dot indicates the number of genes enriched in the corresponding pathway

For further investigating the possible function of the identified miRNAs in regulating fiber initiation, GO and KEGG pathway analyses was carried out for all the target genes of miRNAs expressed in a differential manner at − 3, 0 and 3 DPA. For the target genes, there was significant enrichment of the following 30 terms, 12 biological processes, 13 molecular functions and five cellular component terms (Fig. 7a). The top three biological processes terms were intracellular signal transduction (GO: 0035556) containing 58 genes, signal transduction (GO: 0007165) containing 61 genes, and signaling (GO: 0023052) containing 61 genes. The most significant cellular component terms were nucleus (GO: 0005634) containing 52 genes, intracellular membrane-bounded organelle (GO: 0043231) containing 64 genes, and membrane-bounded organelle (GO: 0043227) containing 64 genes. The most significant molecular function terms were oxidoreductase activity, acting upon a sulfur group of donors, NAD(P) as acceptor (GO: 0016668), protein-disulfide reductase activity (GO: 0047134), and oxidoreductase activity, acting upon a sulfur group of donors (GO: 0016667), those which contained 51 genes.

The KEGG pathway analysis revealed significant enrichment of the following: Plant-pathogen interaction, mRNA surveillance pathway, Glycerolipid metabolism, Circadian rhythm-plant, Plant hormone signal transduction, Endocytosis, and Spliceosome, Selenocompound metabolism (Fig. 7b). GO and KEGG pathway analyses results indicate that exogenous IAA might regulate fiber development through these particular pathways and processes.

### Expression patterns of selected miRNAs and target genes

**Fig. 8:**
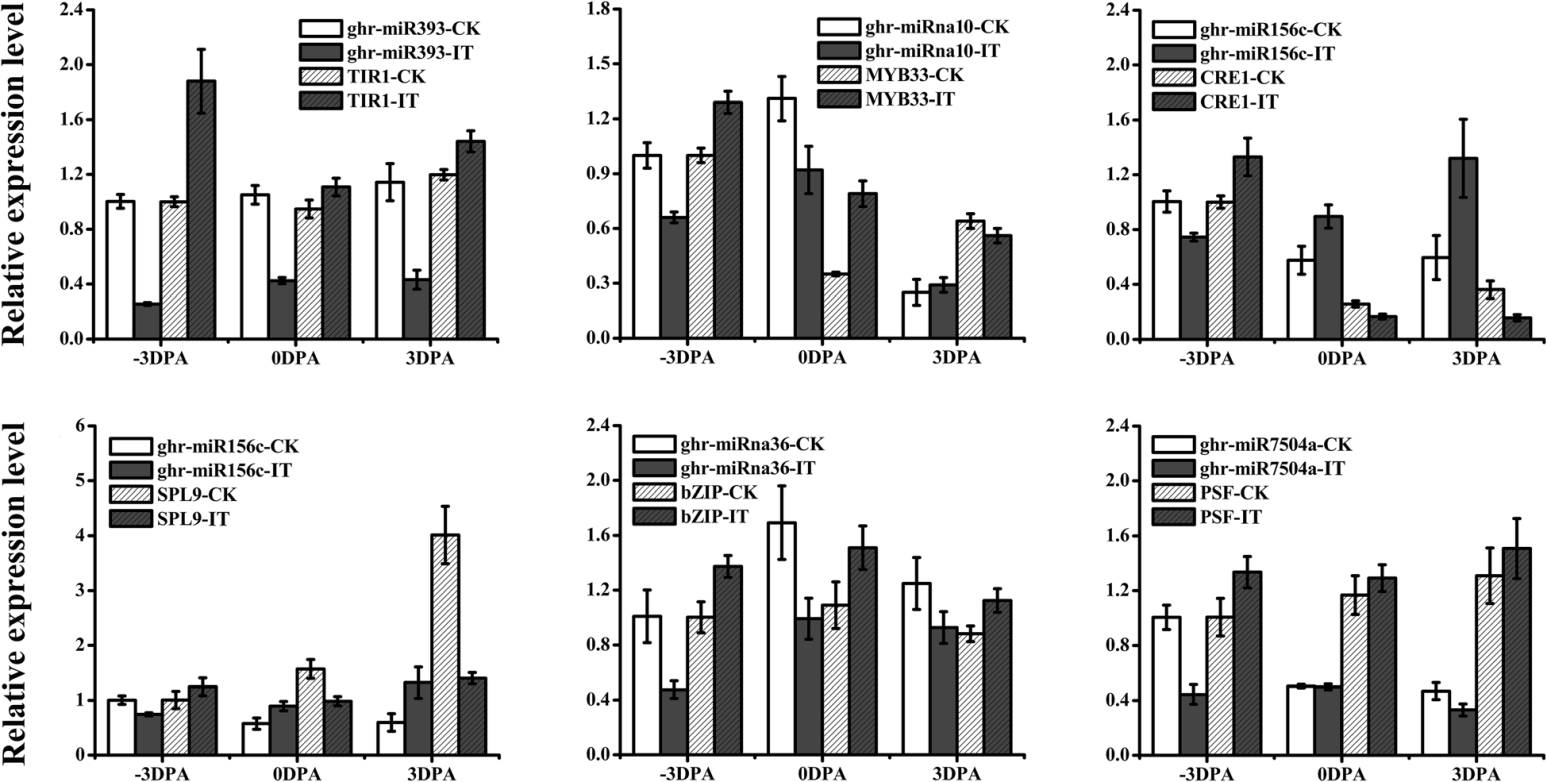
Expression patterns of selected miRNAs and their corresponding target genes. The columns indicated the abundance of miRNAs and target genes for control (CK) and IAA treated (IT) at − 3, 0, and 3 DPA from qRT-PCR results. The data represent the mean values ± SD of the three replicates

Selection and validation of several miRNAs and their target genes related to phytohormones and fiber development were performed by qRT-PCR to analyze their expression patterns. As shown in Fig. 8, the expression levels of all the selected miRNAs, ghr-miR393, ghr-miR10, ghr-miR156c, ghr-miR7504a, and ghr-miR36, were negatively correlated with their target genes. Three phytohormone-related genes were selected, *TIR1*, *MYB33* and *CRE1*. The transcript levels of the auxin receptor gene *TIR1* were significantly higher in IT than in CK at − 3, 0, and 3 DPA. *MYB33*, which encodes a transcription factor that undertakes the positive regulator of ABA, exhibited higher expression levels in IT verses CK at − 3 and 0 DPA and lower expression levels in IT versus CK at 3 DPA. *CRE1*, a cytokinin receptor gene, had higher expression levels in IT versus CK at − 3 DPA and lower expression level in IT at 0 and 3 DPA. Apart from these phytohormone biosynthesis genes, pre-mRNA splicing factor (*PSF*) had higher expression levels in IT than CK at − 3, 0, and 3 DPA. The expression patterns of two fiber-related genes, *SPL9* and *bZIP,* were also investigated. *SPL9*, which can repress trichome formation, had higher expression levels in IT versus CK at − 3 DPA and lower expression levels in IT at 0 and 3 DPA. *bZIP*, a fiber development-related gene, had higher expression in IT than in CK at − 3, 0, and 3 DPA.

## Discussion

Cotton, as the most important natural fiber source for the textile industry, provides income for roughly 100 million families in 150 countries throughout the world [42]. Recently, it was shown that the major phytohormone IAA was a critical regulator during the development of cotton fiber both in vivo and in vitro [10–13]. Moreover, pre- and post-anthesis exogenous IAA treatment was found to contribute to fiber initiation [14]. The present study investigated the regulatory mechanism of the exogenous IAA effect on fiber initiation using small RNA sequencing and profiling.

Cotton fibers emerge from the ovule epidermis at 0 DPA [3, 43]. We observed that both the density and size of fibers on the ovule surface at 0 DPA were significantly increased in IT compared to CK. These findings indicated that exogenous IAA promoted fiber initiation, which is consistent with previous research [14]. Mature fiber quality traits, including FL, FU, FE, and FS, tended to be higher in IT than in CK, although the differences were not significant, the findings suggest that exogenous IAA may have a positive effect on fiber quality, which is particularly relevant for the changes in spinning technology [44]. The significantly higher MIC and LP in IT also suggested that exogenous IAA may contribute to cotton fiber development, and this result is consistent with previous studies [14].

It is well established that endogenous phytohormones critically impact fiber development. In our research, the endogenous IAA content of IT was higher than that of CK after exogenous IAA treatment. It was previously reported that IAA promoted fiber initiation [10, 13]. Therefore, the significantly greater number and size of lint fibers in IT versus CK may have been induced by the increased IAA content in IT. The endogenous GA content was greater than in CK from 0 DPA to 5 DPA, which implies that exogenous IAA promoted endogenous GA content. As previously reported, GA may affect fiber initiation and development [7, 14]. Thus, the increased GA content, which was induced by exogenous application, may have also induced the significantly greater number and size of lint fibers in IT. ABA content was lower in IT than in CK, which suggests that exogenous IAA negatively regulate ABA secretion. ABA decreases the positive effect of GA3 and IAA and has been shown to inhibit the growth of the ovule fiber [9, 10]. On the contrary, ABA played a negative role in fiber development. We therefore hypothesize that exogenous IAA might promote fiber initiation by depressing the ABA biosynthesis. Taken together, the present findings suggest that exogenous IAA affects cotton fiber development in part by regulating the phytohormones network in cotton plants, content of several phytohormones, including increasing the endogenous IAA and GA contents and reducing the endogenous ABA content.

Cotton miRNAs were first identified using a comparative genomic approach [45]. Since then, as deep sequencing technology has provided a powerful high-throughput strategy for miRNA identification, l Numarge numbers of cotton miRNAs have been characterized and implicated in plant growth and resistance to biotic and abiotic stresses.Numerous cotton miRNAs are found to be expressed in a differential manner under salinity, drought and heavy metal stress and had negative correlation to their target genes, indicating a function of these miRNAs in abiotic stress tolerance [46–48]. Moreover, miRNAs are discovered to take part in the response to biotic stresses, such as *Verticillium dahliae* and leafroll dwarf polerovirus in cotton [49–51]. The mechanism of cotton fiber development is complex and still unclear, involving numerous miRNAs with important roles. Several prior studies have identified hundreds of conserved and novel miRNAs related to fiber development [6, 29, 31, 42, 45, 52–57], the first of which were identified and predicted along with their targets by Zhang et al. in 2007. MiR396, miR414, and miR782 have been shown to target genes encoding callous synthase, fiber protein Fb23, and fiber quinine-oxidoreductase, which are closely linked to fiber development [45]. Using small RNA libraries generated from ovules of the fiberless mutant Xu-142w and its wild type control Xu-142 during cotton fiber initiation and development, Kwak et al. identified 22 conserved miRNA families, most of which were significantly differentially expressed such as miR156/157, miR165/166, miR172, miR395, miR397 and miR399. [57]. In 2014, Guan et al. reported that miR828 and miR858 regulated the *MYB2* gene involved in cotton fiber development. *MYB2* could induce trichomes in a no-trichome *Arabidopsis* mutant [58]. So far, no study has researched on the effect of exogenous IAA on the miRNA regulation of fiber initiation. In our present work, deep sequencing of small RNAs was performed for IT and CK cotton ovules and millions of small RNA reads were mapped to the cotton genome. In according to preceding reports in cotton [29] and other plants such as rice [59] and maize [60], 21 and 24 nt miRNAs represented the largest fraction of all the miRNAs. 24 nt miRNAs were reported to be loaded onto AGO4 and showed a preference for sRNAs with 5′ terminal adenine [61, 62]. In our results, adenine at the firstbase contributed the most of all the 24 nt miRNAs. Moreover, uridine was the most nucleotides of all the 21 nt miRNAs, which accorded to the earlier report that miRNAs with firstbase of uridine were usually harbored by AGO1 [62]. Totally, we identified 58 known and 83 novel miRNAs under exogenous IAA treatment during fiber development. At the first position of novel miRNAs, A and U were the prominent nucleotides, in accordance with previous research [31]. Furthermore, 34, 16, and 24 miRNAs were found to be differentially expressed at − 3, 0, and 3 DPA ovules, respectively. It is therefore possible that exogenous IAA promoted fiber initiation through the regulatory activity of these miRNAs on their target genes. MiRNAs with decreasing or increasing expression over time, as determined through cluster analysis result, might also be critical for fiber initiation. As a preliminary step to determine whether any of these miRNAs are indeed critical for cotton fiber development, we identified 357 target genes, which were predicted by the miRNAs expressed in a differential manner, and they were subjected to GO and KEGG analyses. A previously analysis by Xie et al. identified 820 genes from miRNAs related to fiber development, which were associated with 1027 GO terms and 78 KEGG pathways [56]. For the target genes identified in the present study, there was a significant enrichment for 31 GO terms and eight pathways, which might provide some clues about miRNA regulation of fiber development.

The process of cotton fiber development is very complicated, which is related to numerous factors. In the present research, several miRNAs with target genes involved in fiber development were selected to detect their expression patterns. TIR1, an auxin receptor, plays a role in mediating the degradation of auxin/IAA and transcription of auxin-regulated [63]. *TIR1* transcript levels were higher in IT than in CK at − 3, 0, and 3 DPA, which indicated that exogenous IAA affected the auxin metabolism in the cotton plants. The finding further suggested that exogenous IAA promoted fiber initiation through the regulation of auxin metabolism in plants. MiR393, which regulates *TIR1*, was identified in the analysis, and its expression was lower in IT than in CK at − 3, 0, and 3 DPA, consistent with its negative regulatory effect on *TIR1*. As such, miR393 probably played a role in the regulating the development of cotton fiber.*MYB33*, a transcription gene that positively regulates ABA response, is a target gene of ghr-miR319 [64]. In our research, ghr-miR10 showed high homologies with ghr-miR319, which was also predicted to regulate the *MYB33,* exhibited lower expression levels in CK than in IT at − 3 and 0 DPA and higher expression levels at 3 DPA. The expression of *MYB33* was the opposite. In accordance with a reported research, our results showed that the ABA content was synchronous with the expression of ghr-miR319 [65, 66]. However, the expression pattern of *MYB33* was opposite to ABA content. We speculate that there might be a balance among miRna11 cleavage, *MYB33* expression and ABA content, similar to that described in a previous report [65] and that these three factors all participate in the regulation of fiber initiation. CRE1, a cytokinin receptor, was identified previously [67]. In our research, *CRE1* transcripts, targeted by ghr-miR156c, were more abundant in IT than in CK at − 3 DPA and less abundant at 0 and − 3 DPA, which was opposite to the expression pattern of ghr-miR156c. This result was consistent with a previous report that cytokinins stimulate fiber initiations before flowering and inhibit fiber growth after flowering [68]. The finding also suggests that the positive effect of exogenous IAA on fiber initiation may involve the regulation of cytokinins metabolism. Ghr-miR156, one of the most conserved miRNAs, also targets *SPL9,* which controls trichome distribution in *Arabidopsis* [69]*,* a process akin to cotton fiber development. In our analysis, *SPL9* transcripts were lower at 0 and 3 DPA in IT, suggesting that the down regulated expression of *SPL9* might promote the cotton fiber initiation. Another transcription factor of interest, *bZIP*, regulates fiber development in *Gossypium barbadense* L*.* [70]. Its expression was higher in IT at − 3 and 0 DPA and lower at 3 DPA, indicating that *bZIP* might have a positive effect on fiber initiation. An opposite expression pattern was found for the corresponding miRNA, ghr-miR36. We also investigated the expression pattern of the pre-mRNA splicing factor gene *PSF* [53], which is regulated by ghr-miR7504a. Its expression was higher at − 3, 0, and 3 DPA in IT, suggesting that increased mRNA maturation may be required for more biochemical processes under exogenous IAA treatment. Altogether, our analysis identified many candidate miRNAs potentially involved in cotton fiber initiation.

## Conclusion

Cotton serves as a foremost fiber plant worldwide, and the development process of cotton fiber is complex. Here, we found that exogenous IAA application on flower buds before anthesis affected the IAA, GA and ABA contents in the ovule and increased fiber initiation and early development at the fiber initiation stage. Using small RNA sequencing, altogether 58 known miRNAs and 83 novel miRNAs with target genes were identified. The differential expression patterns of certain miRNAs and GO and KEGG analyses further illustrated that miRNAs were very important in the regulation of fiber initiation. These results lay the foundation for investigating the complex regulatory network of fiber development, which offer clues for fiber melioration using molecular strategies.

## Additional files

Additional file 1: **Table S1**. All primers used in this study. (DOCX 14 kb)

Additional file 2: **Table S2**. Names and sequences of the identified known miRNAs in six libraries. (XLSX 10 kb)

Additional file 3: **Table S3**. Names and sequences of the identified novel miRNAs in six libraries. (XLSX 11 kb)

Additional file 4: Structures of all the identified novel miRNAs. (ZIP 1005 kb)

Additional file 5: **Table S4**. MiRNAs and the corresponding target genes. (XLSX 30 kb)
