# Supplementary material for: Identification and profiling of upland cotton microRNAs at fiber initiation stage under exogenous IAA application
Source: BMC Genomics. 2019 May 28;20:421. doi: 10.1186/s12864-019-5760-8 (PMC6537205; doi:10.1186/s12864-019-5760-8)
Supplement: Supplementary file 1 — Table S1. All primers used in this study. (DOCX 14 kb) [file 12864_2019_5760_MOESM1_ESM.docx]

**Table S1: All primers used in this study.**

| **miRNA** | **Sequence** |
| --- | --- |
| **ghr-miR393-F** | TCCAAAGGGATCGCATTGAT |
| **ghr-miR10-F** | GCGTTGGACTGAAGGGAGCTC |
| **ghr-miR156c-F** | TGTCAGAAGAGAGTGAGCAC |
| **ghr-miR36-F** | TTTTTCCATGTCACAAAGATGC |
| **ghr-miR7504a-F** | TATGAAACTGTGATTCCACGTCAT |
| **TIR1-F** | TTCTTGCGCTGTGAGCTTCGCA |
| **TIR1-R** | CATGTCGAACCTTGGCCCTGCA |
| **MYB33-F** | CACCAACCAGCATAGCAGAGTCA |
| **MYB33-R** | TCCACAGTTTTCCCATTCT |
| **CRE-F** | TGGACAGATGTCTATGAACGGCA |
| **CRE-R** | TGCCACATCCGAGAAATGCTAT |
| **SPL-F** | GGTCTGGAACGCCGGTTCTGC |
| **SPL-R** | GGCGTCTCGCATTGTGGTCG |
| **bZIP-F** | GTTTGGGAACAAGGACTGC |
| **bZIP-R** | GGATTTTGCAGCTCTCCCACT |
| **PSF-F** | TTATGAGTGGGGACGGGTTA |
| **PSF-R** | CACAGTTTCACCTTCTTCCAA |
| **U6-F** | GGGGACATCCGATAAAATTGG |
| **U6-R** | CATTTCTCGATTTGTGCGTGTC |
| **UBQ7-F** | GAAGGCATTCCACCTGACCAAC |
| **UBQ7-R** | CTTGACCTTCTTCTTCTTGTGCTTG |
