# Supplementary figures and images for: Identification and profiling of upland cotton microRNAs at fiber initiation stage under exogenous IAA application
Source: BMC Genomics. 2019 May 28;20:421. doi: 10.1186/s12864-019-5760-8 (PMC6537205; doi:10.1186/s12864-019-5760-8)

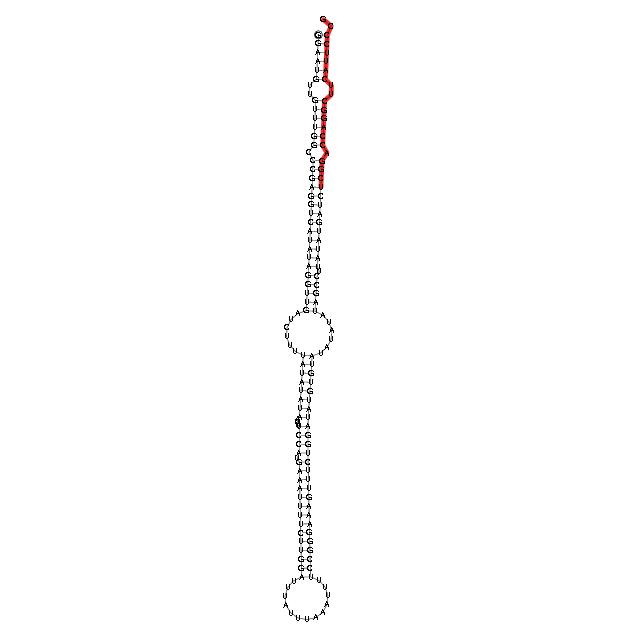

Supplement: Supplementary file 4 — Structures of all the identified novel miRNAs. (ZIP 1005 kb) [file 12864_2019_5760_MOESM4_ESM.zip › Additional file 4/ghr-miR1.jpg]

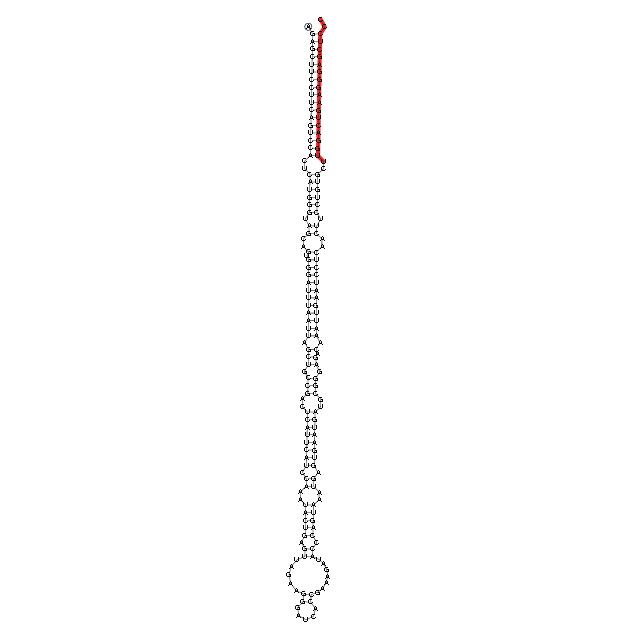

Supplement: Supplementary file 4 — Structures of all the identified novel miRNAs. (ZIP 1005 kb) [file 12864_2019_5760_MOESM4_ESM.zip › Additional file 4/ghr-miR10.jpg]

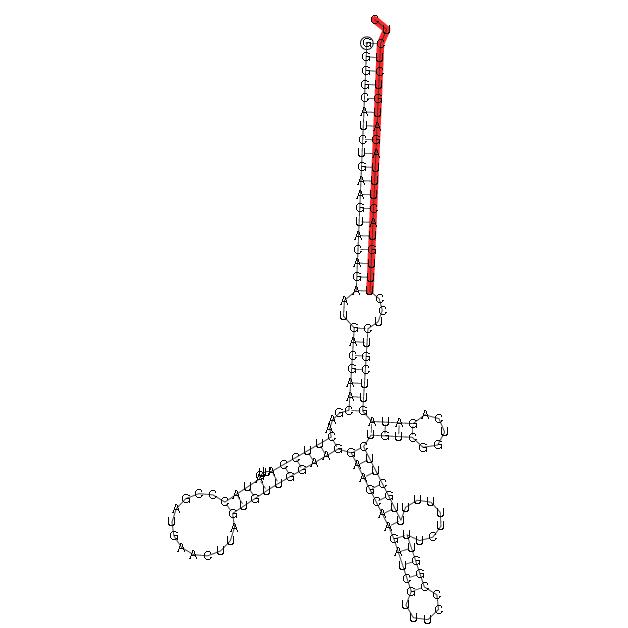

Supplement: Supplementary file 4 — Structures of all the identified novel miRNAs. (ZIP 1005 kb) [file 12864_2019_5760_MOESM4_ESM.zip › Additional file 4/ghr-miR11.jpg]

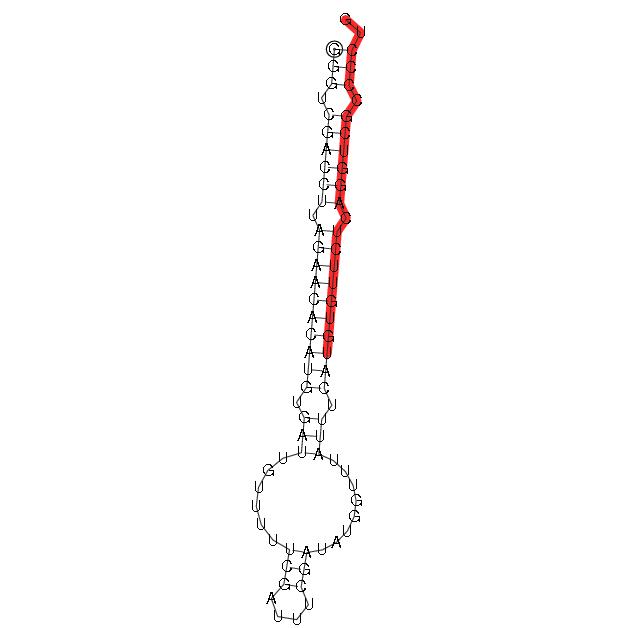

Supplement: Supplementary file 4 — Structures of all the identified novel miRNAs. (ZIP 1005 kb) [file 12864_2019_5760_MOESM4_ESM.zip › Additional file 4/ghr-miR12.jpg]

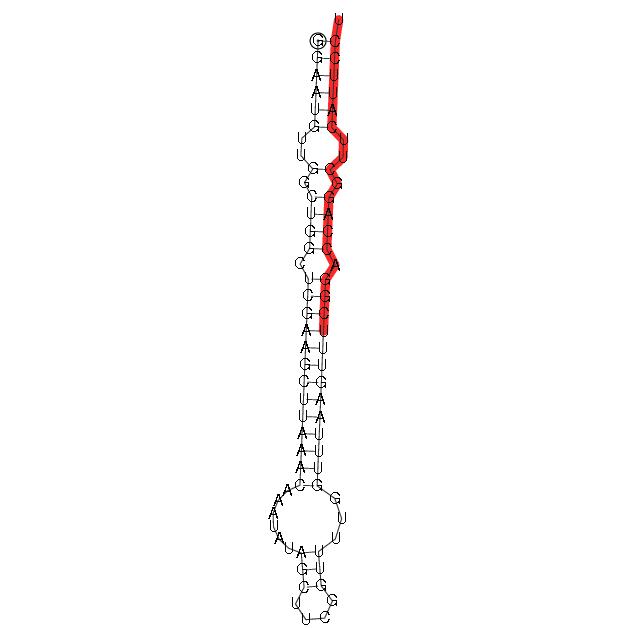

Supplement: Supplementary file 4 — Structures of all the identified novel miRNAs. (ZIP 1005 kb) [file 12864_2019_5760_MOESM4_ESM.zip › Additional file 4/ghr-miR13.jpg]

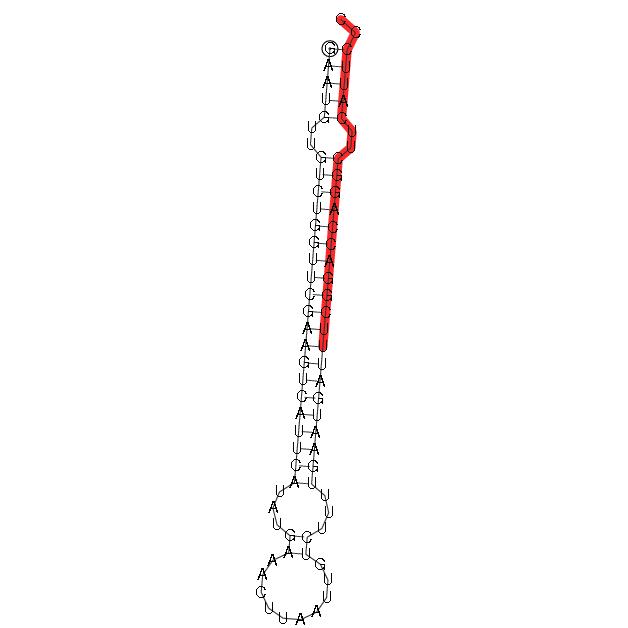

Supplement: Supplementary file 4 — Structures of all the identified novel miRNAs. (ZIP 1005 kb) [file 12864_2019_5760_MOESM4_ESM.zip › Additional file 4/ghr-miR14.jpg]

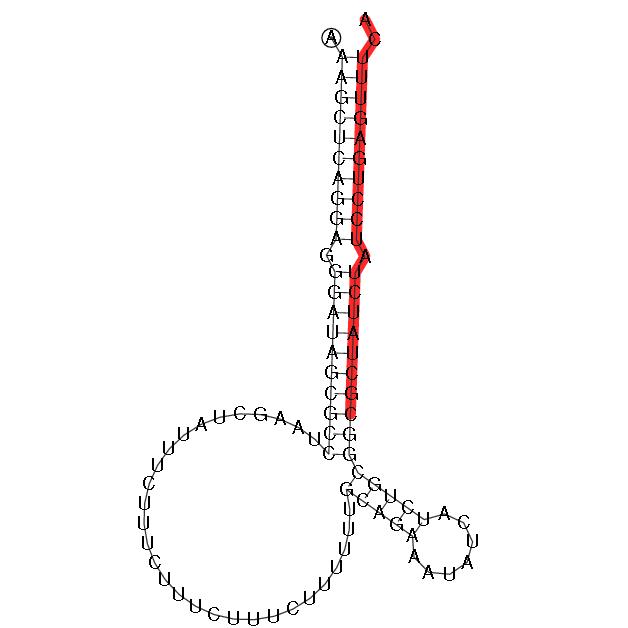

Supplement: Supplementary file 4 — Structures of all the identified novel miRNAs. (ZIP 1005 kb) [file 12864_2019_5760_MOESM4_ESM.zip › Additional file 4/ghr-miR15.jpg]

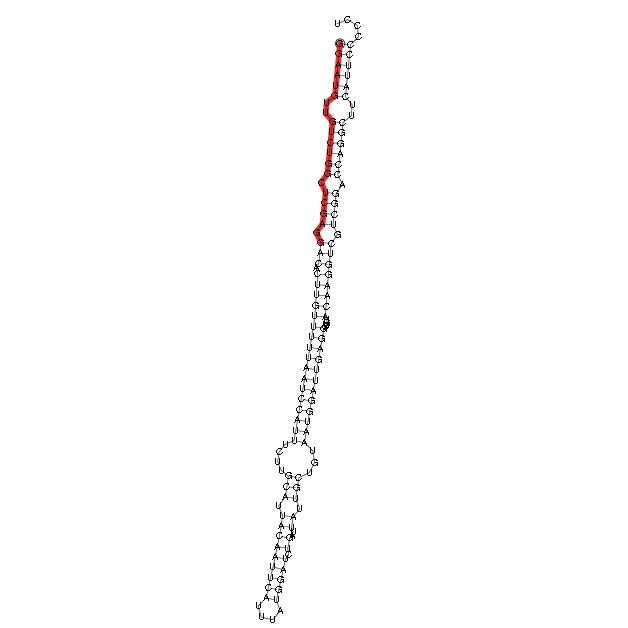

Supplement: Supplementary file 4 — Structures of all the identified novel miRNAs. (ZIP 1005 kb) [file 12864_2019_5760_MOESM4_ESM.zip › Additional file 4/ghr-miR16.jpg]

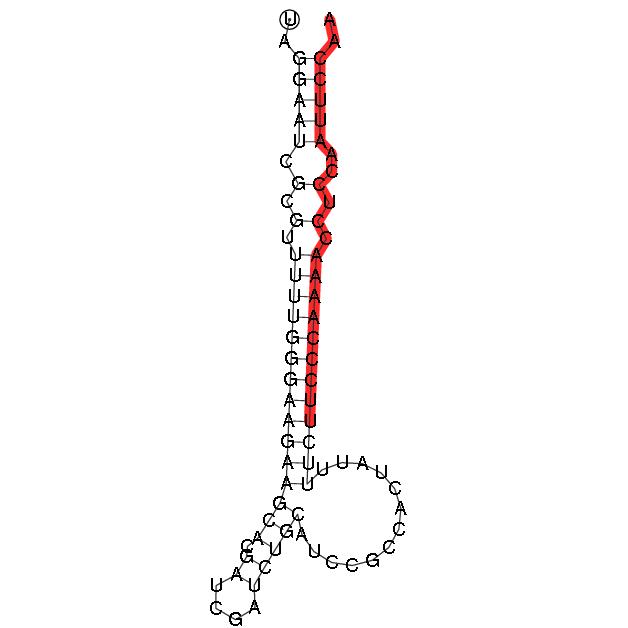

Supplement: Supplementary file 4 — Structures of all the identified novel miRNAs. (ZIP 1005 kb) [file 12864_2019_5760_MOESM4_ESM.zip › Additional file 4/ghr-miR17.jpg]

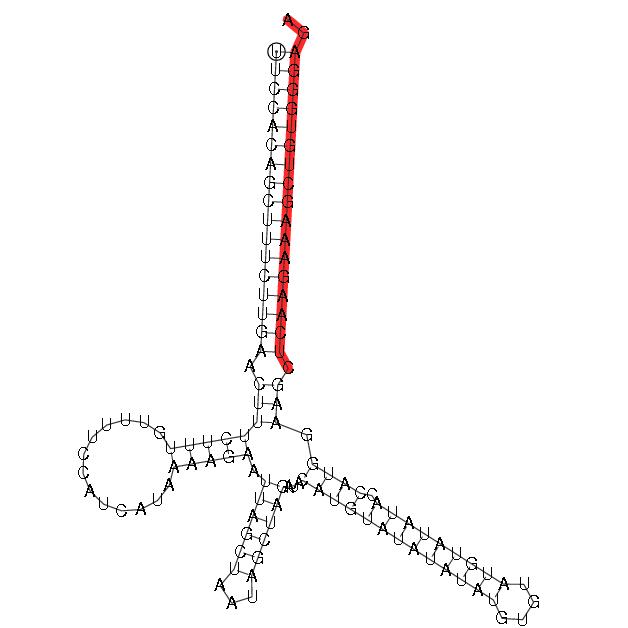

Supplement: Supplementary file 4 — Structures of all the identified novel miRNAs. (ZIP 1005 kb) [file 12864_2019_5760_MOESM4_ESM.zip › Additional file 4/ghr-miR18.jpg]

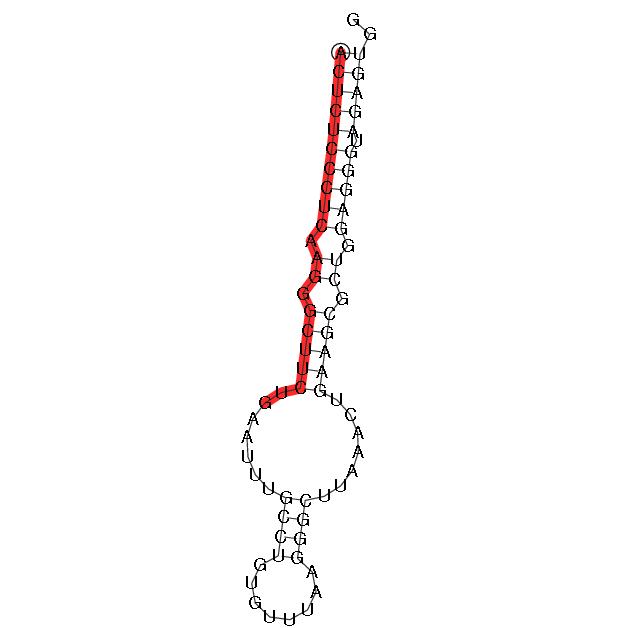

Supplement: Supplementary file 4 — Structures of all the identified novel miRNAs. (ZIP 1005 kb) [file 12864_2019_5760_MOESM4_ESM.zip › Additional file 4/ghr-miR19.jpg]

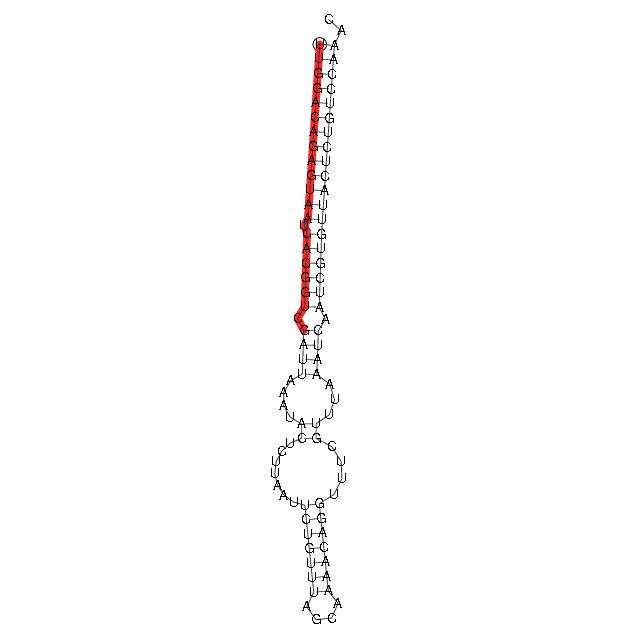

Supplement: Supplementary file 4 — Structures of all the identified novel miRNAs. (ZIP 1005 kb) [file 12864_2019_5760_MOESM4_ESM.zip › Additional file 4/ghr-miR2.jpg]

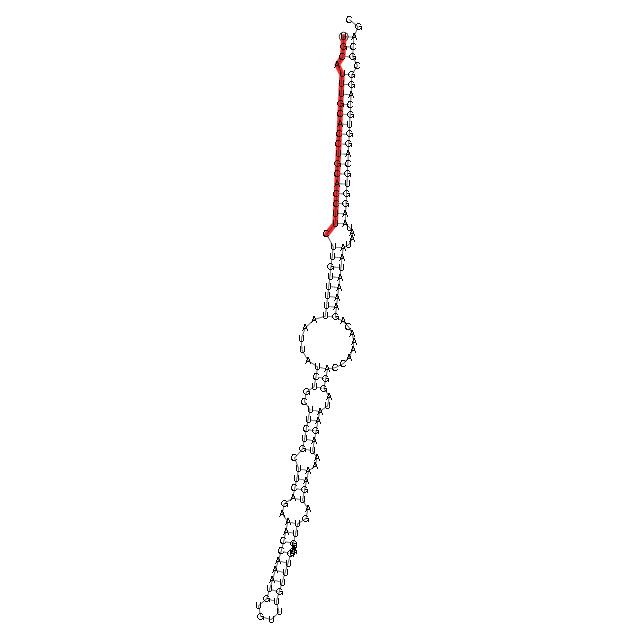

Supplement: Supplementary file 4 — Structures of all the identified novel miRNAs. (ZIP 1005 kb) [file 12864_2019_5760_MOESM4_ESM.zip › Additional file 4/ghr-miR20.jpg]

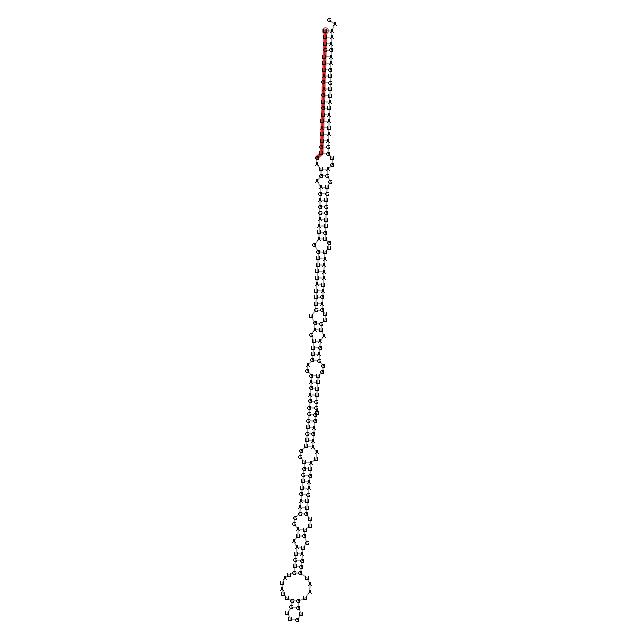

Supplement: Supplementary file 4 — Structures of all the identified novel miRNAs. (ZIP 1005 kb) [file 12864_2019_5760_MOESM4_ESM.zip › Additional file 4/ghr-miR21.jpg]

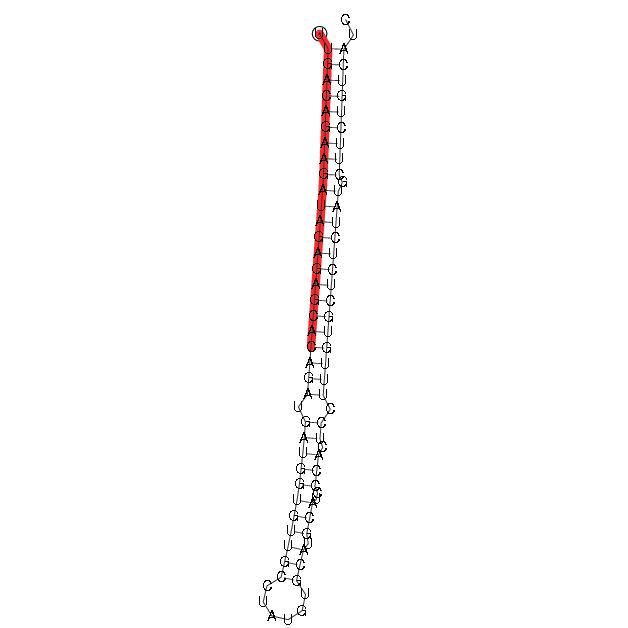

Supplement: Supplementary file 4 — Structures of all the identified novel miRNAs. (ZIP 1005 kb) [file 12864_2019_5760_MOESM4_ESM.zip › Additional file 4/ghr-miR22.jpg]

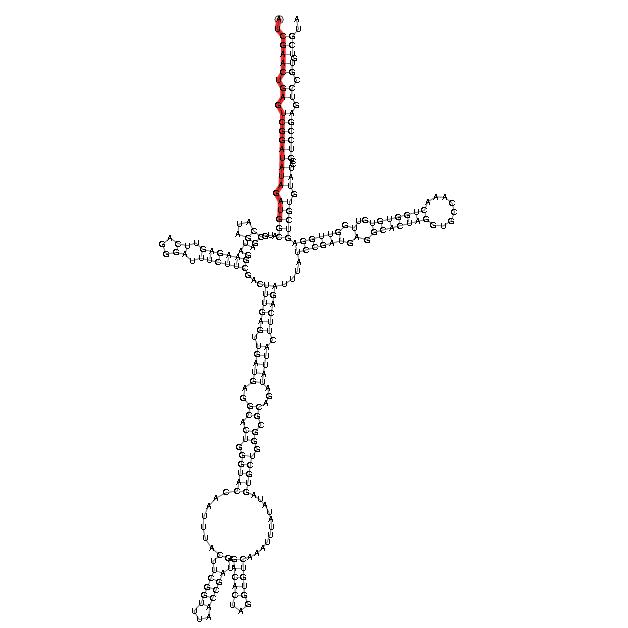

Supplement: Supplementary file 4 — Structures of all the identified novel miRNAs. (ZIP 1005 kb) [file 12864_2019_5760_MOESM4_ESM.zip › Additional file 4/ghr-miR23.jpg]

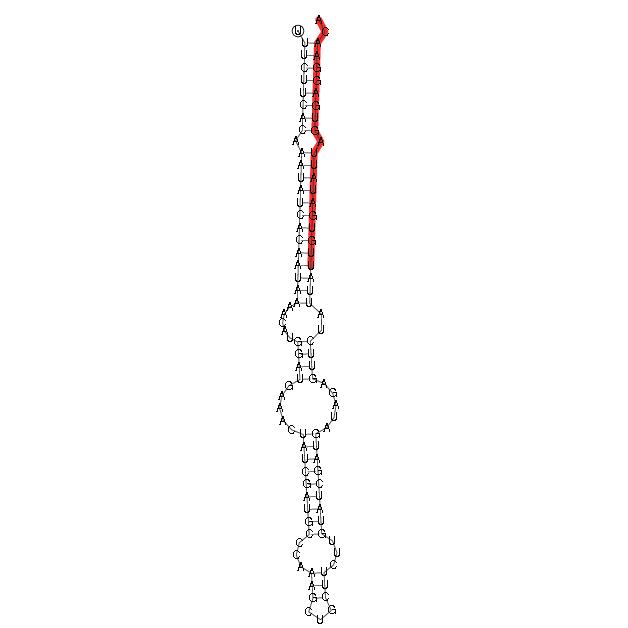

Supplement: Supplementary file 4 — Structures of all the identified novel miRNAs. (ZIP 1005 kb) [file 12864_2019_5760_MOESM4_ESM.zip › Additional file 4/ghr-miR24.jpg]

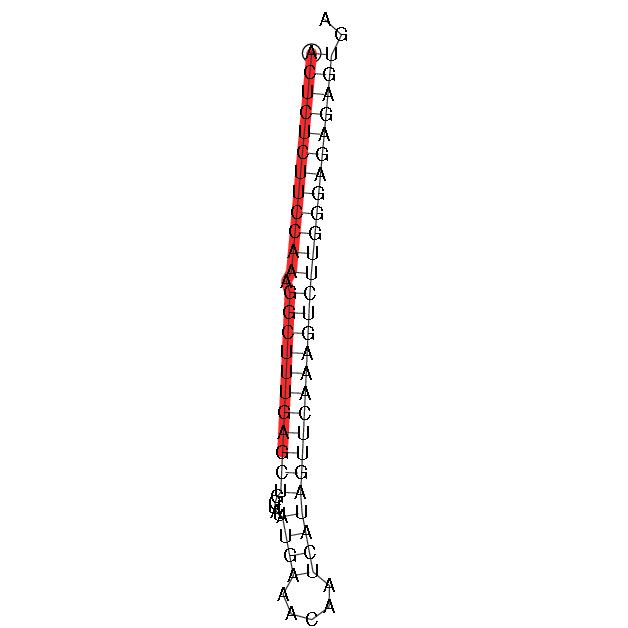

Supplement: Supplementary file 4 — Structures of all the identified novel miRNAs. (ZIP 1005 kb) [file 12864_2019_5760_MOESM4_ESM.zip › Additional file 4/ghr-miR25.jpg]

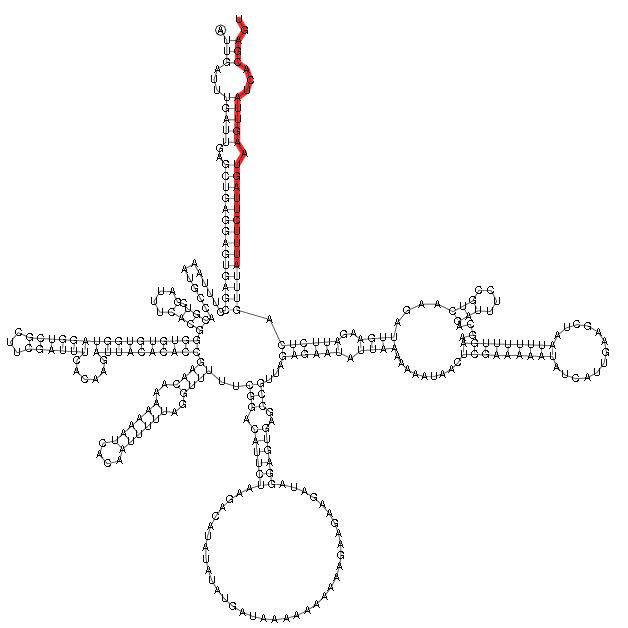

Supplement: Supplementary file 4 — Structures of all the identified novel miRNAs. (ZIP 1005 kb) [file 12864_2019_5760_MOESM4_ESM.zip › Additional file 4/ghr-miR26.jpg]

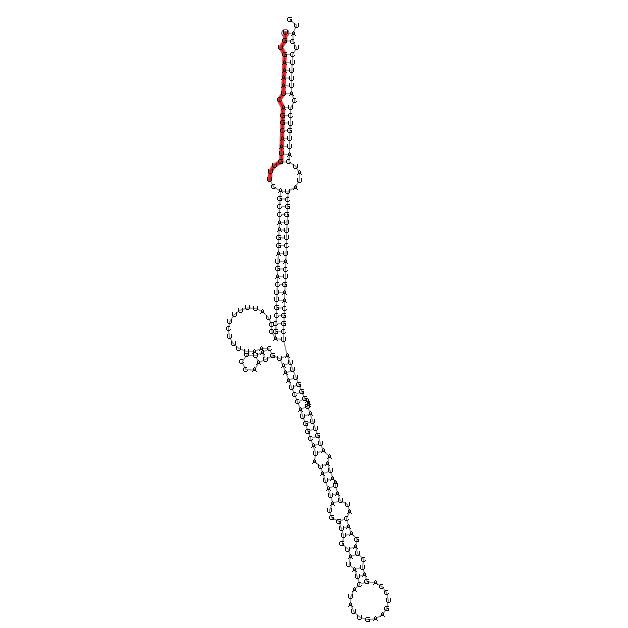

Supplement: Supplementary file 4 — Structures of all the identified novel miRNAs. (ZIP 1005 kb) [file 12864_2019_5760_MOESM4_ESM.zip › Additional file 4/ghr-miR27.jpg]

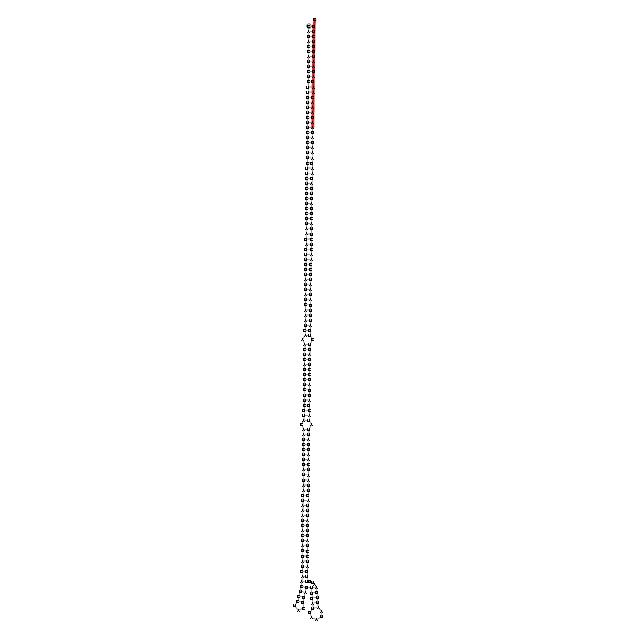

Supplement: Supplementary file 4 — Structures of all the identified novel miRNAs. (ZIP 1005 kb) [file 12864_2019_5760_MOESM4_ESM.zip › Additional file 4/ghr-miR28.jpg]

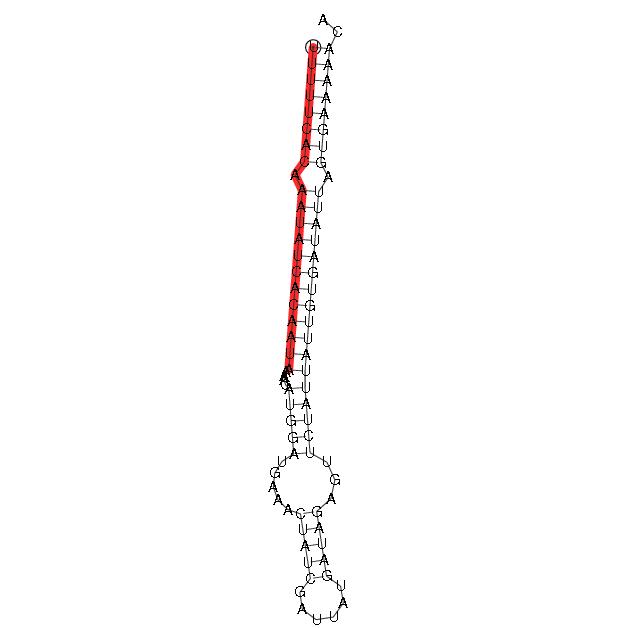

Supplement: Supplementary file 4 — Structures of all the identified novel miRNAs. (ZIP 1005 kb) [file 12864_2019_5760_MOESM4_ESM.zip › Additional file 4/ghr-miR29.jpg]

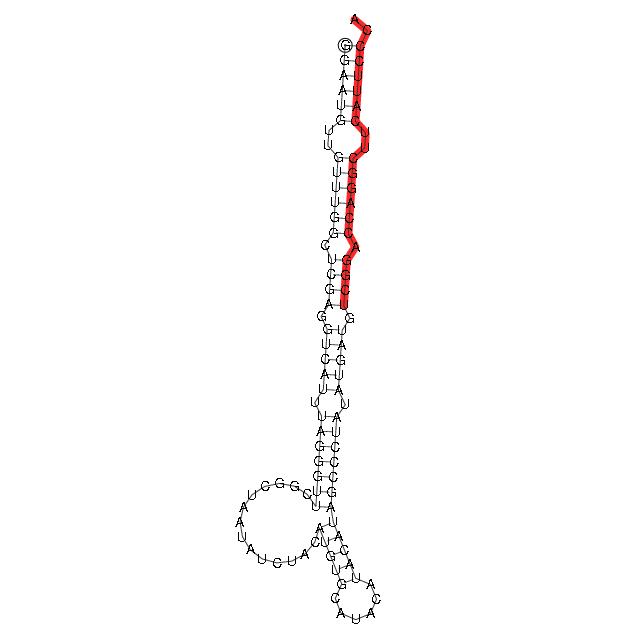

Supplement: Supplementary file 4 — Structures of all the identified novel miRNAs. (ZIP 1005 kb) [file 12864_2019_5760_MOESM4_ESM.zip › Additional file 4/ghr-miR3.jpg]

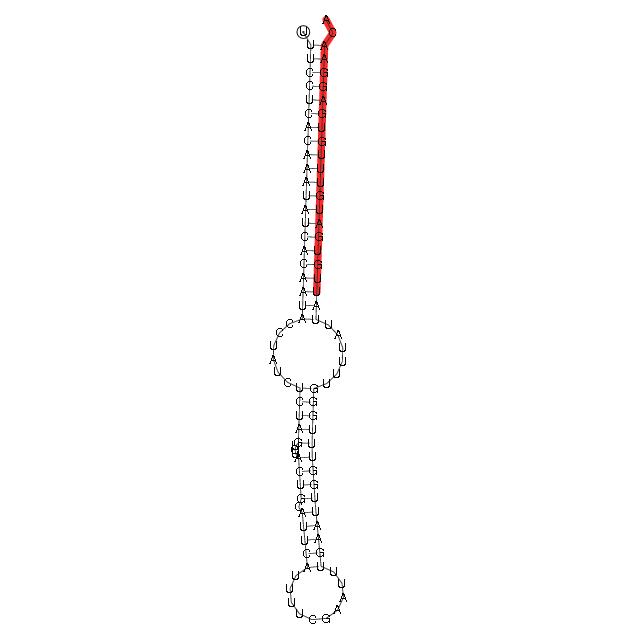

Supplement: Supplementary file 4 — Structures of all the identified novel miRNAs. (ZIP 1005 kb) [file 12864_2019_5760_MOESM4_ESM.zip › Additional file 4/ghr-miR30.jpg]

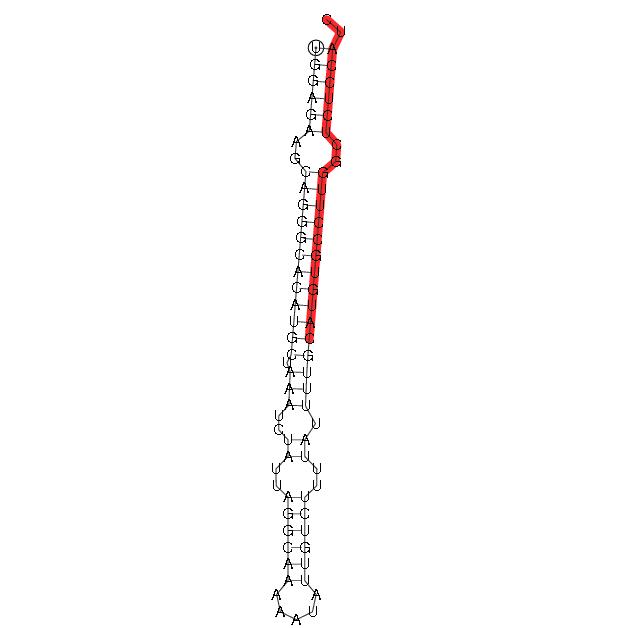

Supplement: Supplementary file 4 — Structures of all the identified novel miRNAs. (ZIP 1005 kb) [file 12864_2019_5760_MOESM4_ESM.zip › Additional file 4/ghr-miR31.jpg]

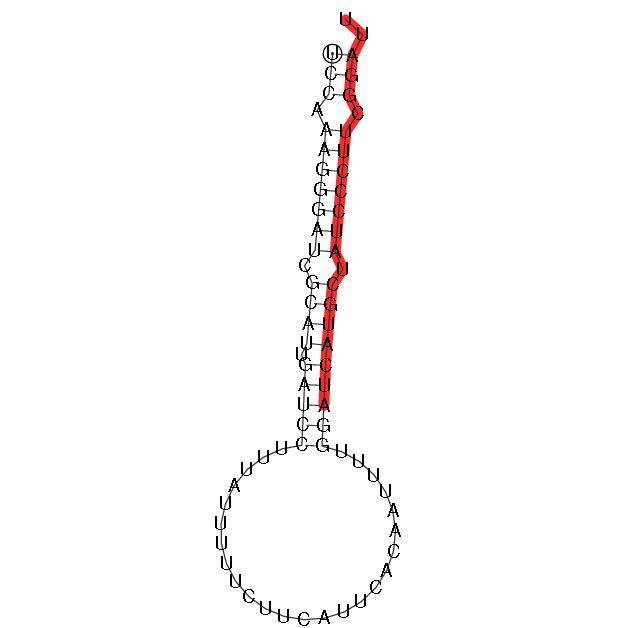

Supplement: Supplementary file 4 — Structures of all the identified novel miRNAs. (ZIP 1005 kb) [file 12864_2019_5760_MOESM4_ESM.zip › Additional file 4/ghr-miR32.jpg]

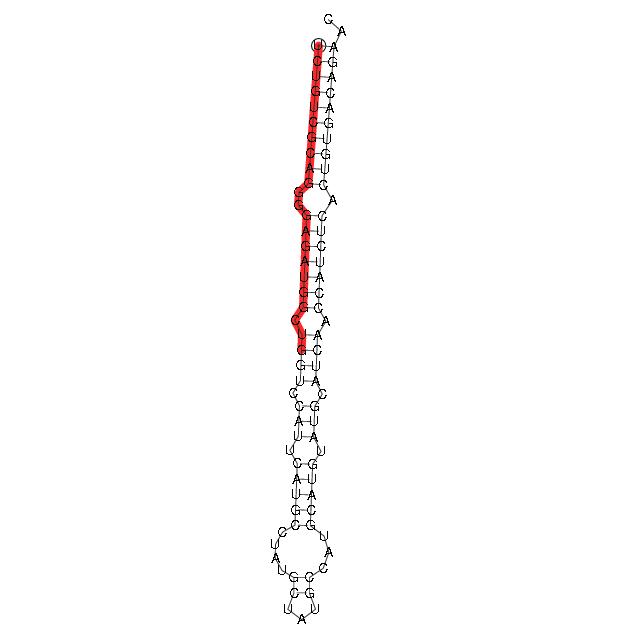

Supplement: Supplementary file 4 — Structures of all the identified novel miRNAs. (ZIP 1005 kb) [file 12864_2019_5760_MOESM4_ESM.zip › Additional file 4/ghr-miR33.jpg]

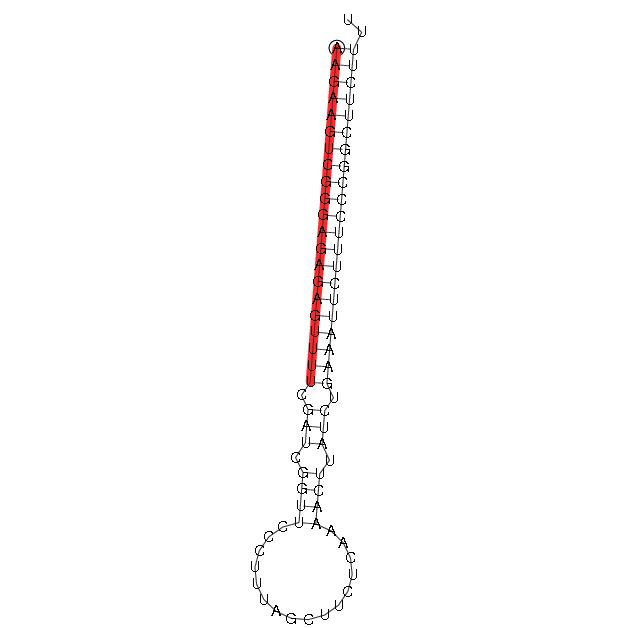

Supplement: Supplementary file 4 — Structures of all the identified novel miRNAs. (ZIP 1005 kb) [file 12864_2019_5760_MOESM4_ESM.zip › Additional file 4/ghr-miR34.jpg]

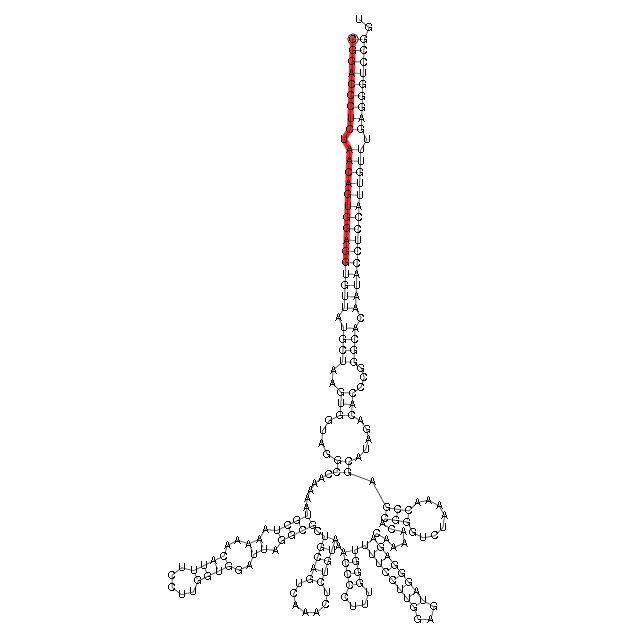

Supplement: Supplementary file 4 — Structures of all the identified novel miRNAs. (ZIP 1005 kb) [file 12864_2019_5760_MOESM4_ESM.zip › Additional file 4/ghr-miR35.jpg]

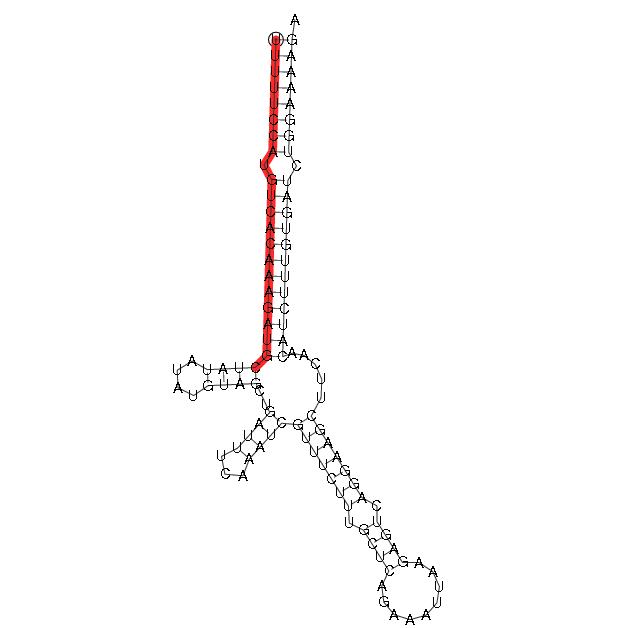

Supplement: Supplementary file 4 — Structures of all the identified novel miRNAs. (ZIP 1005 kb) [file 12864_2019_5760_MOESM4_ESM.zip › Additional file 4/ghr-miR36.jpg]

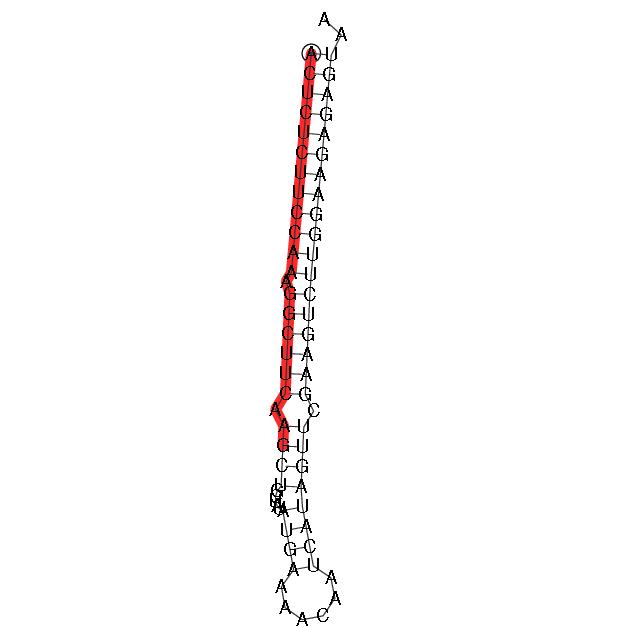

Supplement: Supplementary file 4 — Structures of all the identified novel miRNAs. (ZIP 1005 kb) [file 12864_2019_5760_MOESM4_ESM.zip › Additional file 4/ghr-miR37.jpg]

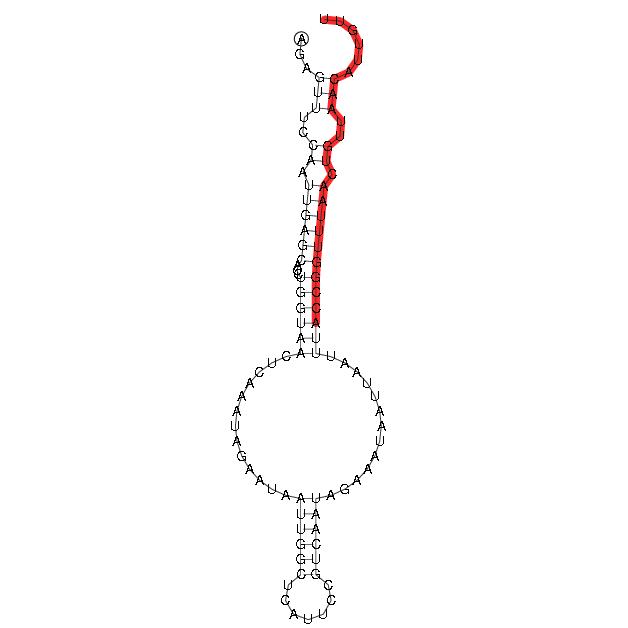

Supplement: Supplementary file 4 — Structures of all the identified novel miRNAs. (ZIP 1005 kb) [file 12864_2019_5760_MOESM4_ESM.zip › Additional file 4/ghr-miR38.jpg]

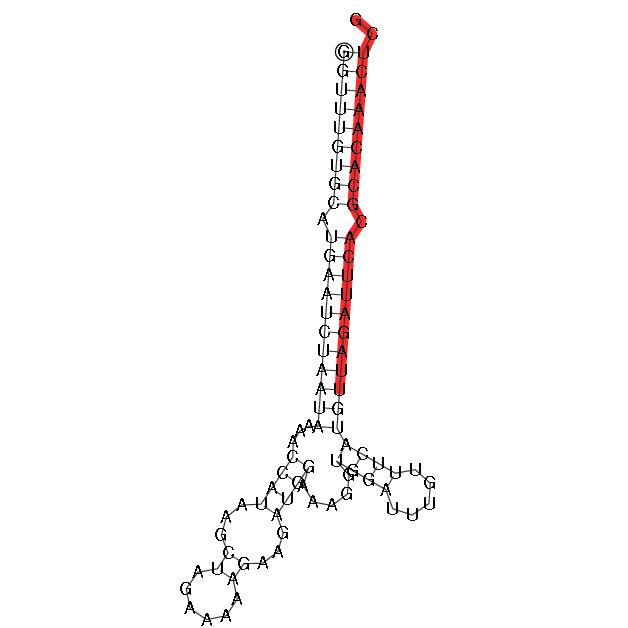

Supplement: Supplementary file 4 — Structures of all the identified novel miRNAs. (ZIP 1005 kb) [file 12864_2019_5760_MOESM4_ESM.zip › Additional file 4/ghr-miR39.jpg]

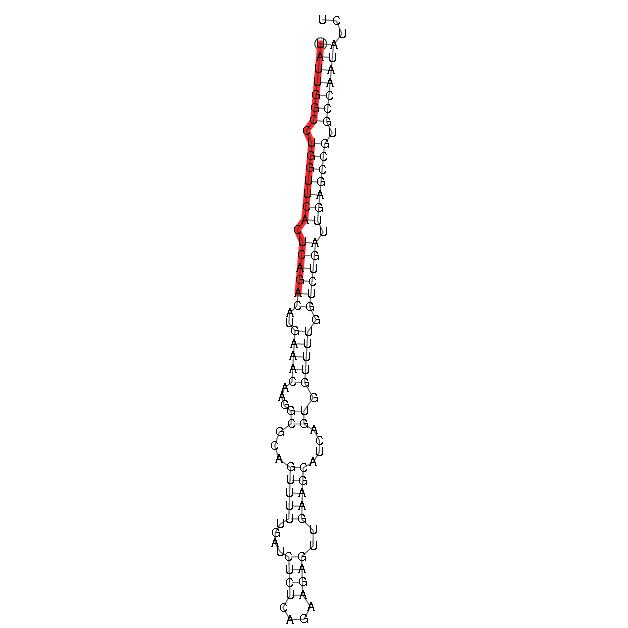

Supplement: Supplementary file 4 — Structures of all the identified novel miRNAs. (ZIP 1005 kb) [file 12864_2019_5760_MOESM4_ESM.zip › Additional file 4/ghr-miR4.jpg]

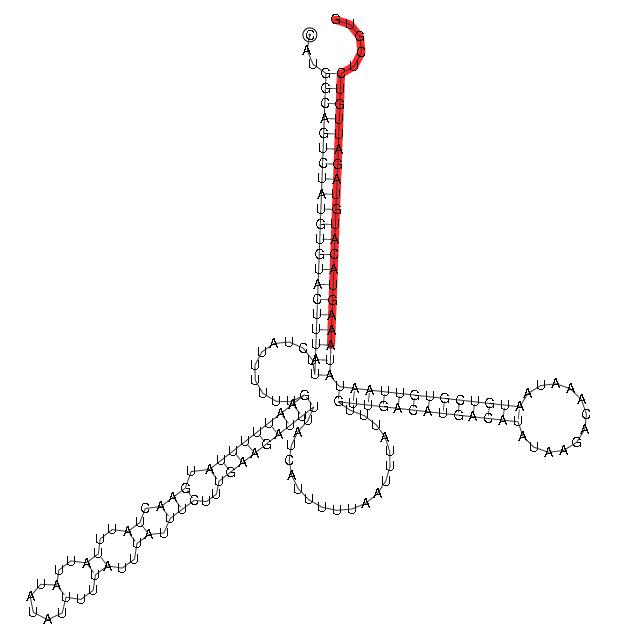

Supplement: Supplementary file 4 — Structures of all the identified novel miRNAs. (ZIP 1005 kb) [file 12864_2019_5760_MOESM4_ESM.zip › Additional file 4/ghr-miR40.jpg]

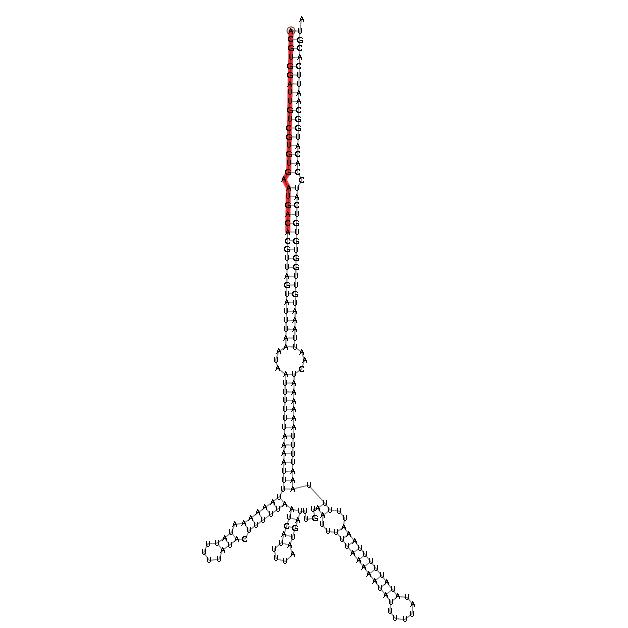

Supplement: Supplementary file 4 — Structures of all the identified novel miRNAs. (ZIP 1005 kb) [file 12864_2019_5760_MOESM4_ESM.zip › Additional file 4/ghr-miR41.jpg]

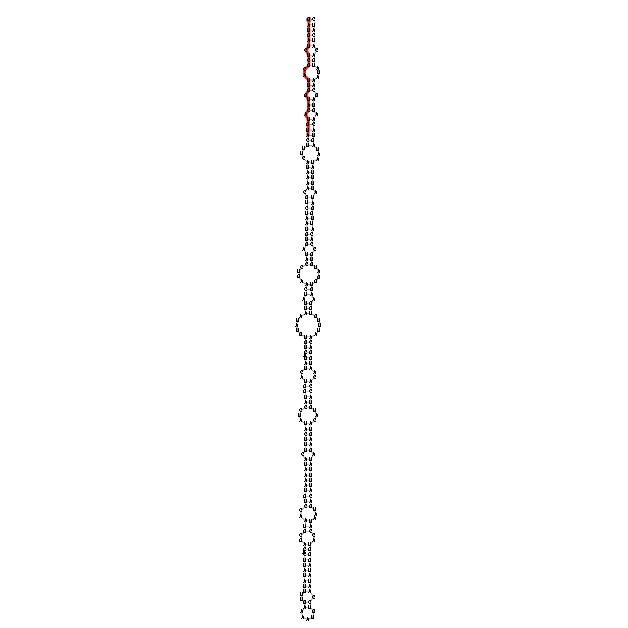

Supplement: Supplementary file 4 — Structures of all the identified novel miRNAs. (ZIP 1005 kb) [file 12864_2019_5760_MOESM4_ESM.zip › Additional file 4/ghr-miR42.jpg]

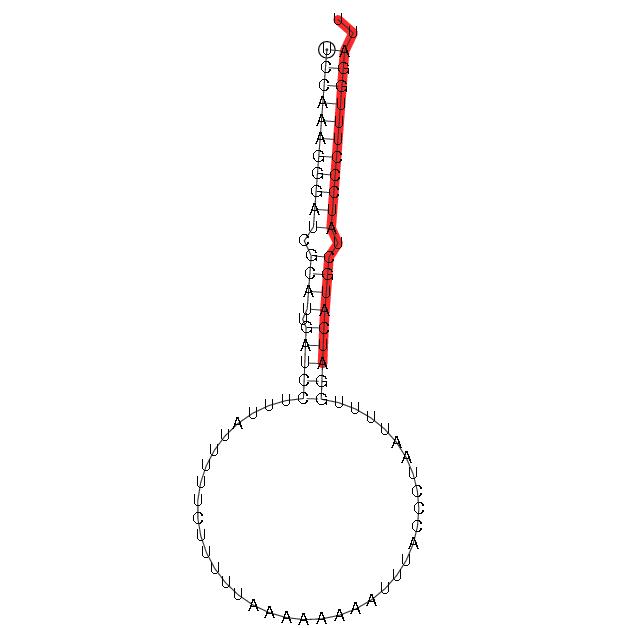

Supplement: Supplementary file 4 — Structures of all the identified novel miRNAs. (ZIP 1005 kb) [file 12864_2019_5760_MOESM4_ESM.zip › Additional file 4/ghr-miR43.jpg]

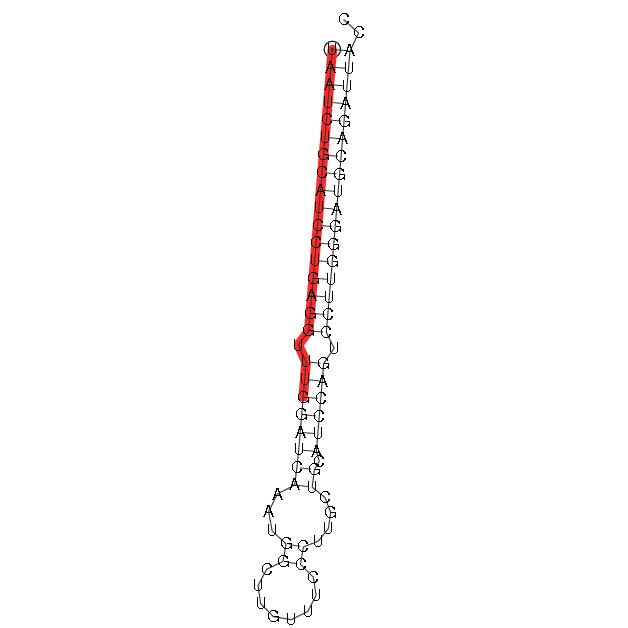

Supplement: Supplementary file 4 — Structures of all the identified novel miRNAs. (ZIP 1005 kb) [file 12864_2019_5760_MOESM4_ESM.zip › Additional file 4/ghr-miR44.jpg]

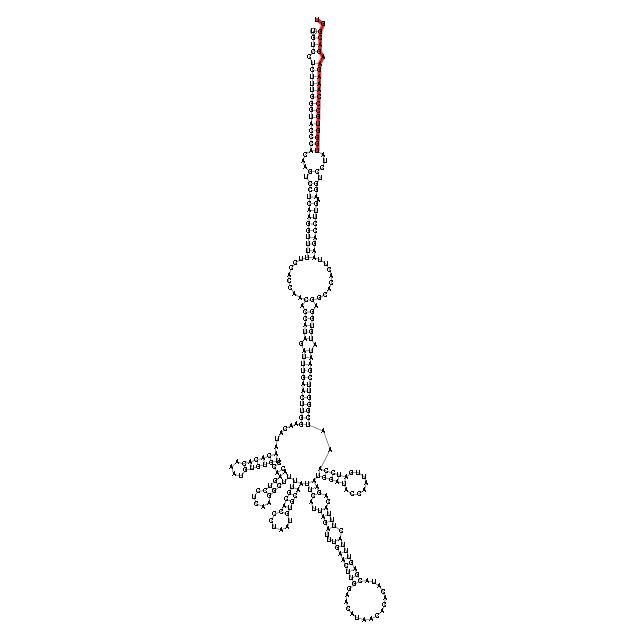

Supplement: Supplementary file 4 — Structures of all the identified novel miRNAs. (ZIP 1005 kb) [file 12864_2019_5760_MOESM4_ESM.zip › Additional file 4/ghr-miR45.jpg]

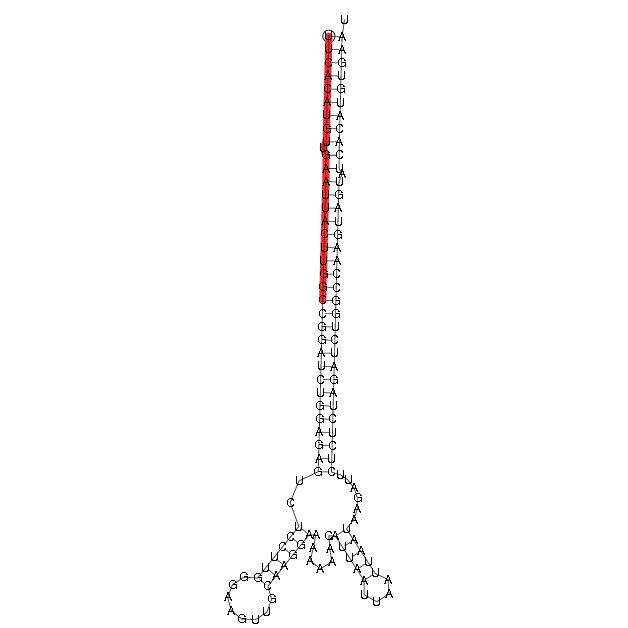

Supplement: Supplementary file 4 — Structures of all the identified novel miRNAs. (ZIP 1005 kb) [file 12864_2019_5760_MOESM4_ESM.zip › Additional file 4/ghr-miR46.jpg]

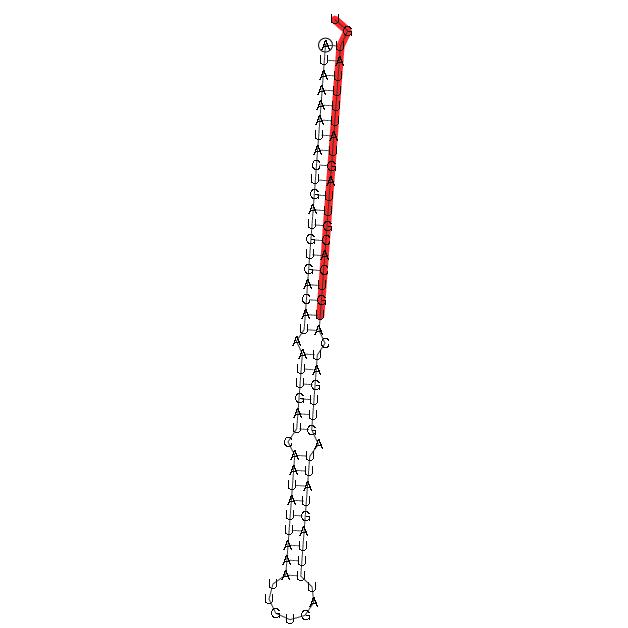

Supplement: Supplementary file 4 — Structures of all the identified novel miRNAs. (ZIP 1005 kb) [file 12864_2019_5760_MOESM4_ESM.zip › Additional file 4/ghr-miR47.jpg]

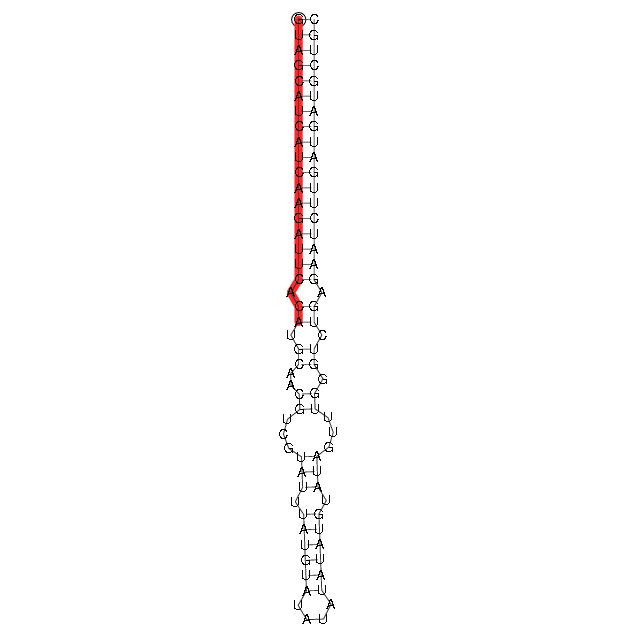

Supplement: Supplementary file 4 — Structures of all the identified novel miRNAs. (ZIP 1005 kb) [file 12864_2019_5760_MOESM4_ESM.zip › Additional file 4/ghr-miR48.jpg]

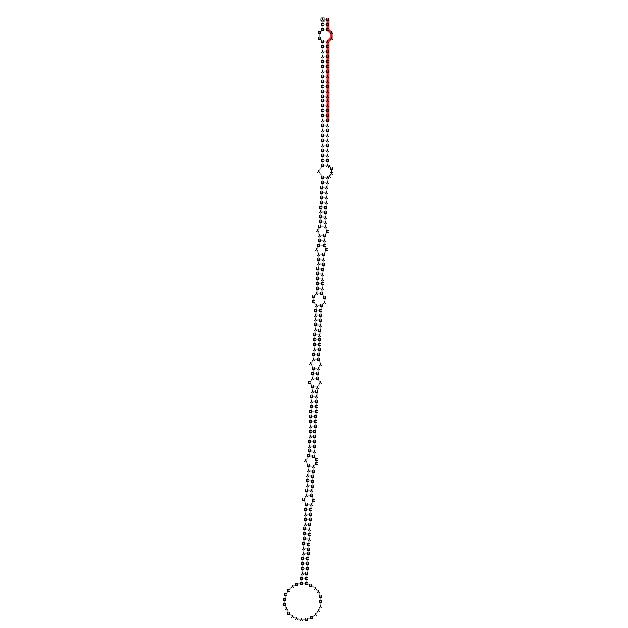

Supplement: Supplementary file 4 — Structures of all the identified novel miRNAs. (ZIP 1005 kb) [file 12864_2019_5760_MOESM4_ESM.zip › Additional file 4/ghr-miR49.jpg]

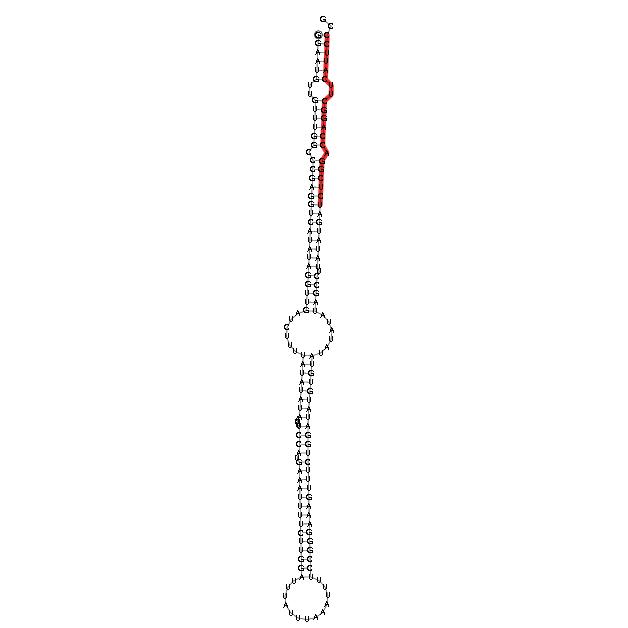

Supplement: Supplementary file 4 — Structures of all the identified novel miRNAs. (ZIP 1005 kb) [file 12864_2019_5760_MOESM4_ESM.zip › Additional file 4/ghr-miR5-1.jpg]

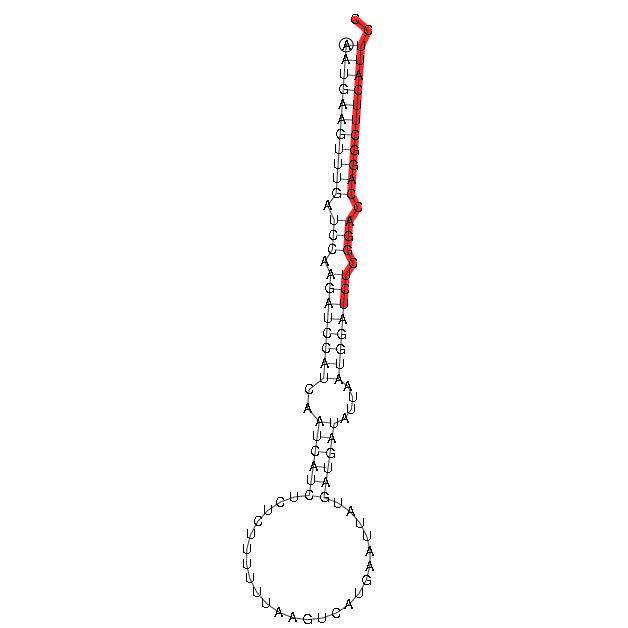

Supplement: Supplementary file 4 — Structures of all the identified novel miRNAs. (ZIP 1005 kb) [file 12864_2019_5760_MOESM4_ESM.zip › Additional file 4/ghr-miR5-2.jpg]

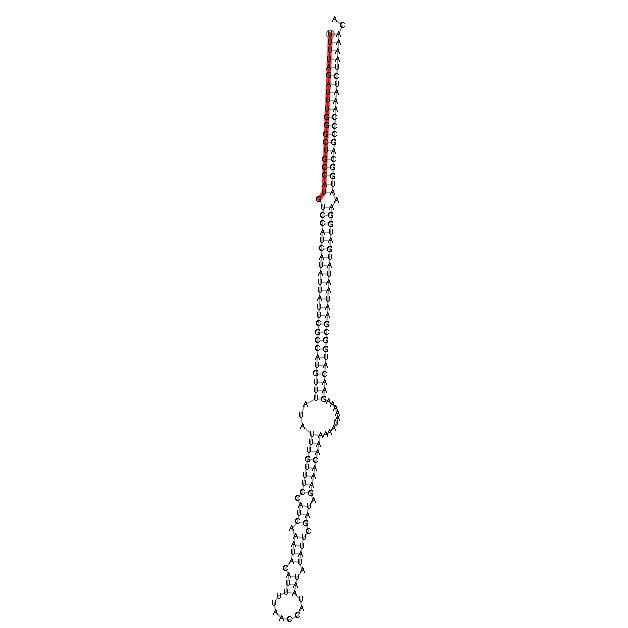

Supplement: Supplementary file 4 — Structures of all the identified novel miRNAs. (ZIP 1005 kb) [file 12864_2019_5760_MOESM4_ESM.zip › Additional file 4/ghr-miR50.jpg]

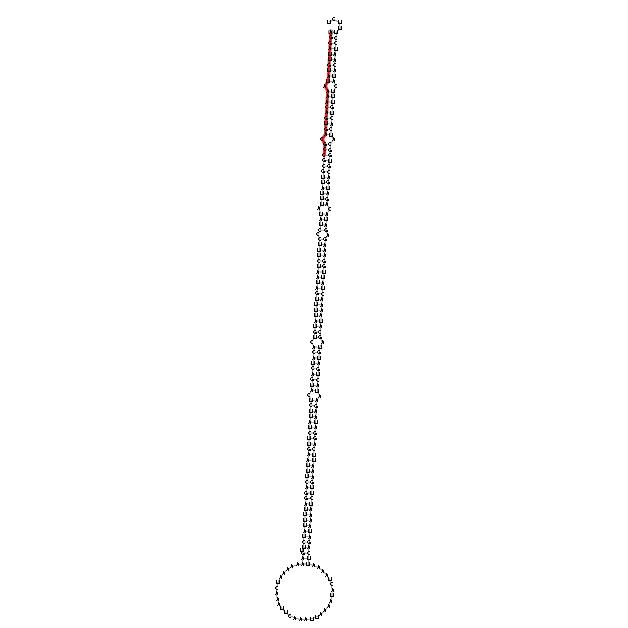

Supplement: Supplementary file 4 — Structures of all the identified novel miRNAs. (ZIP 1005 kb) [file 12864_2019_5760_MOESM4_ESM.zip › Additional file 4/ghr-miR51.jpg]

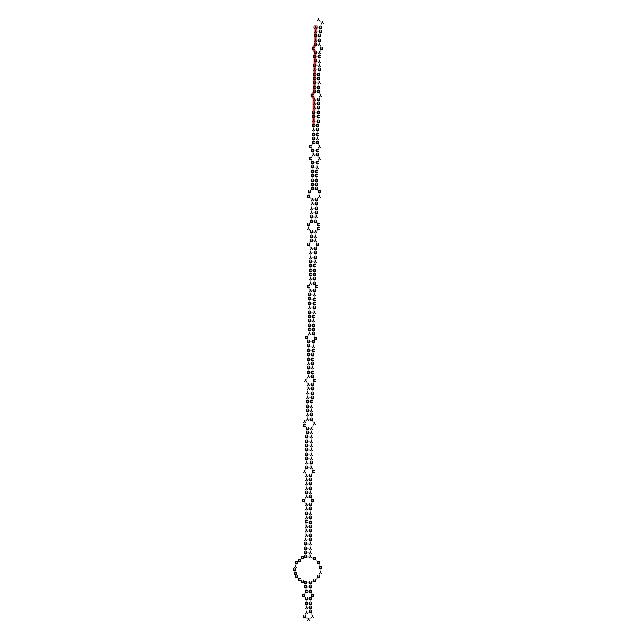

Supplement: Supplementary file 4 — Structures of all the identified novel miRNAs. (ZIP 1005 kb) [file 12864_2019_5760_MOESM4_ESM.zip › Additional file 4/ghr-miR52.jpg]

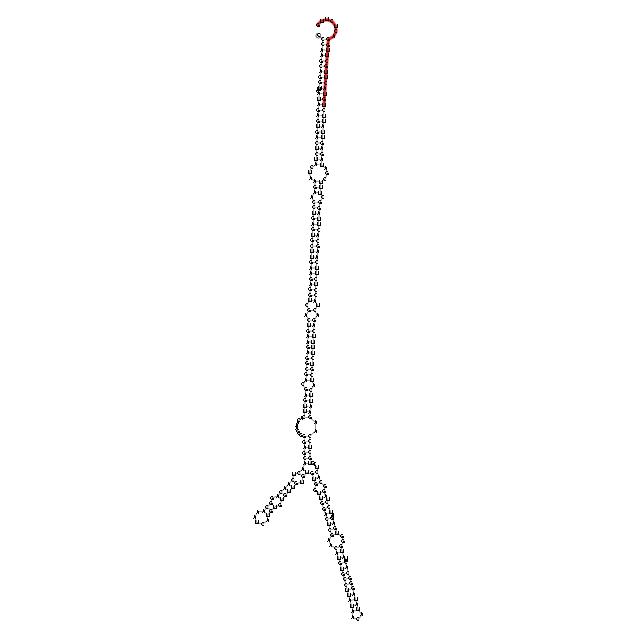

Supplement: Supplementary file 4 — Structures of all the identified novel miRNAs. (ZIP 1005 kb) [file 12864_2019_5760_MOESM4_ESM.zip › Additional file 4/ghr-miR53.jpg]

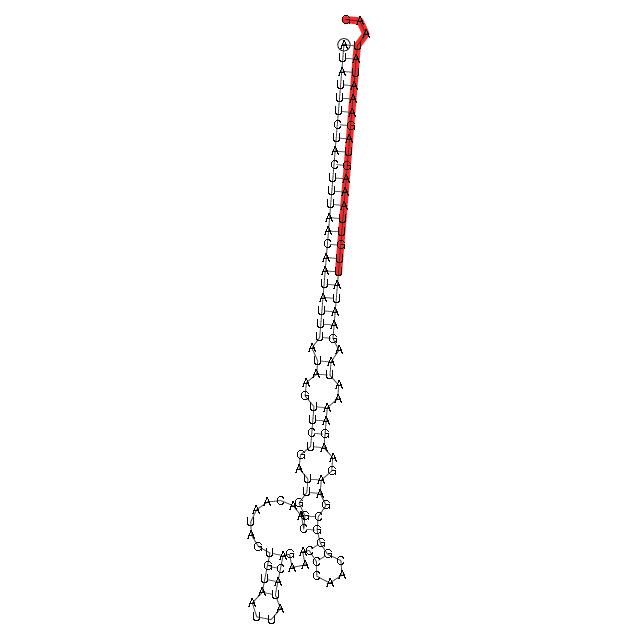

Supplement: Supplementary file 4 — Structures of all the identified novel miRNAs. (ZIP 1005 kb) [file 12864_2019_5760_MOESM4_ESM.zip › Additional file 4/ghr-miR54.jpg]

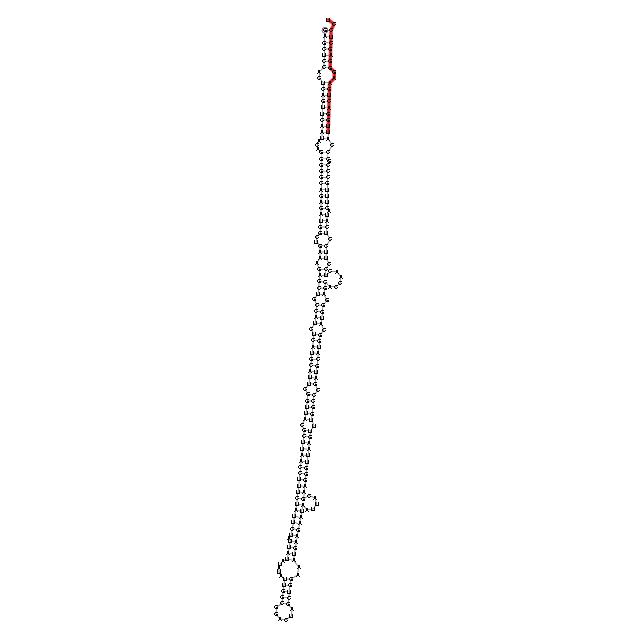

Supplement: Supplementary file 4 — Structures of all the identified novel miRNAs. (ZIP 1005 kb) [file 12864_2019_5760_MOESM4_ESM.zip › Additional file 4/ghr-miR55.jpg]

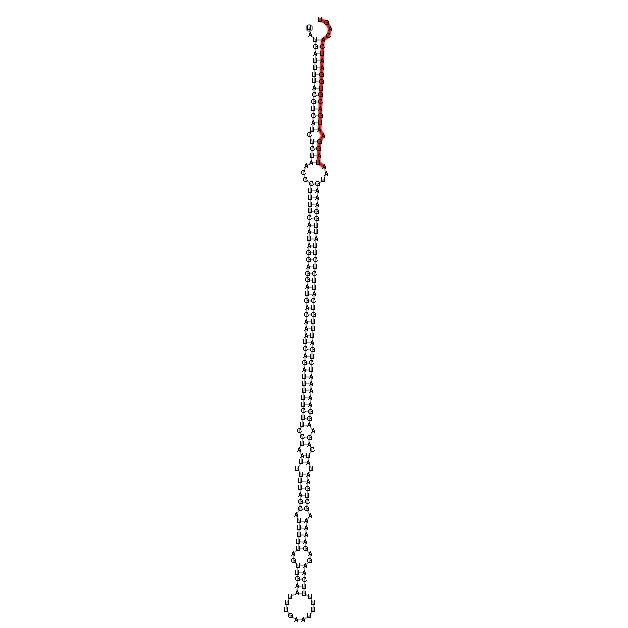

Supplement: Supplementary file 4 — Structures of all the identified novel miRNAs. (ZIP 1005 kb) [file 12864_2019_5760_MOESM4_ESM.zip › Additional file 4/ghr-miR56.jpg]

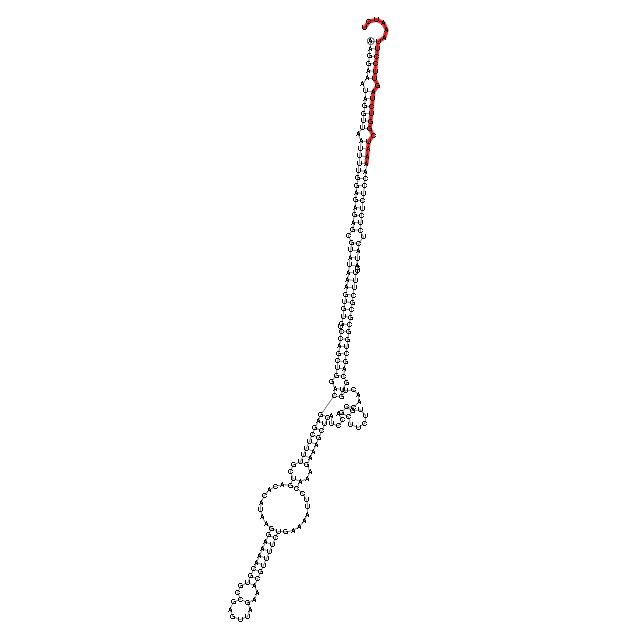

Supplement: Supplementary file 4 — Structures of all the identified novel miRNAs. (ZIP 1005 kb) [file 12864_2019_5760_MOESM4_ESM.zip › Additional file 4/ghr-miR57.jpg]

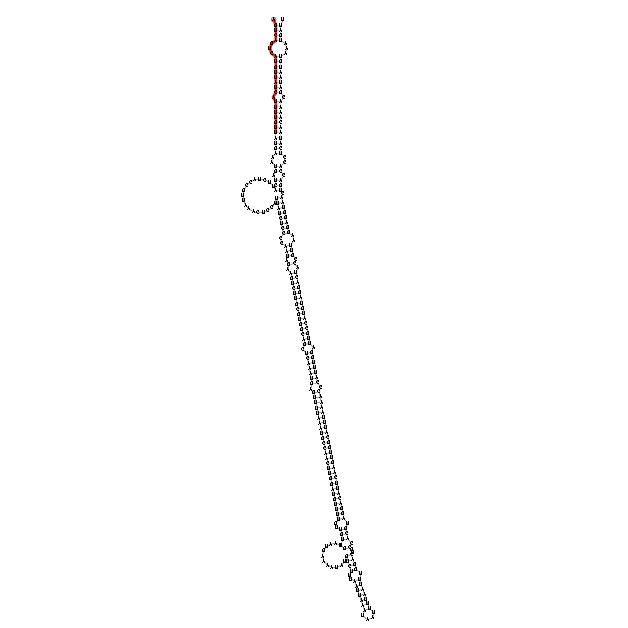

Supplement: Supplementary file 4 — Structures of all the identified novel miRNAs. (ZIP 1005 kb) [file 12864_2019_5760_MOESM4_ESM.zip › Additional file 4/ghr-miR58.jpg]

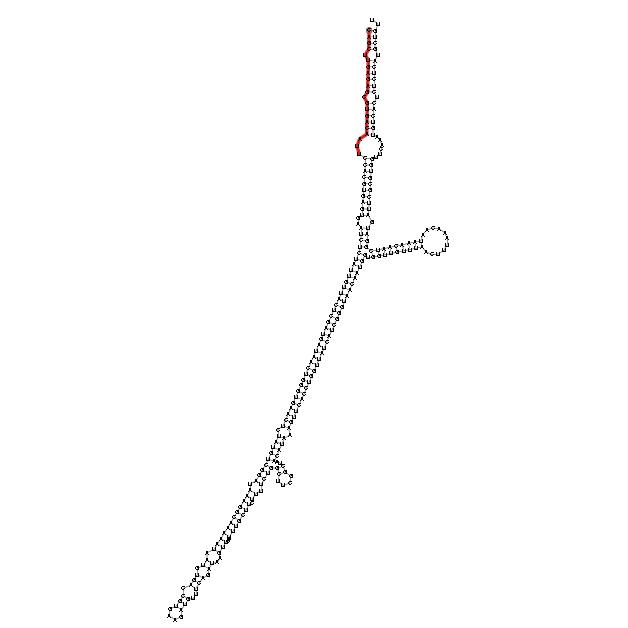

Supplement: Supplementary file 4 — Structures of all the identified novel miRNAs. (ZIP 1005 kb) [file 12864_2019_5760_MOESM4_ESM.zip › Additional file 4/ghr-miR59.jpg]

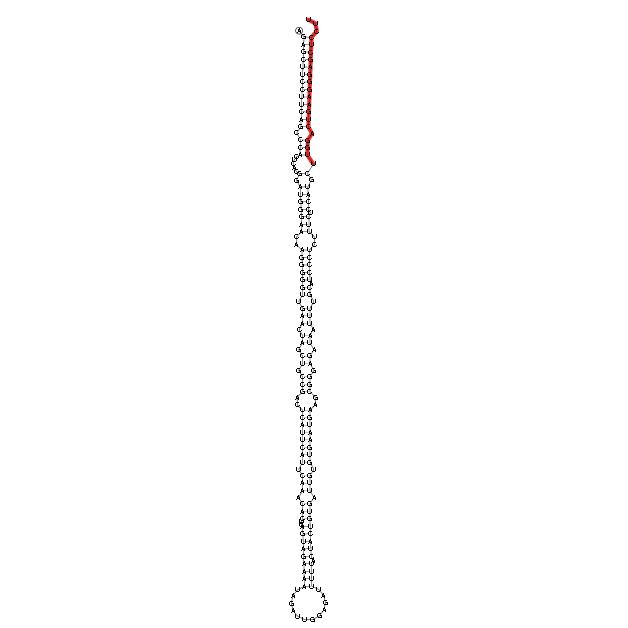

Supplement: Supplementary file 4 — Structures of all the identified novel miRNAs. (ZIP 1005 kb) [file 12864_2019_5760_MOESM4_ESM.zip › Additional file 4/ghr-miR6.jpg]

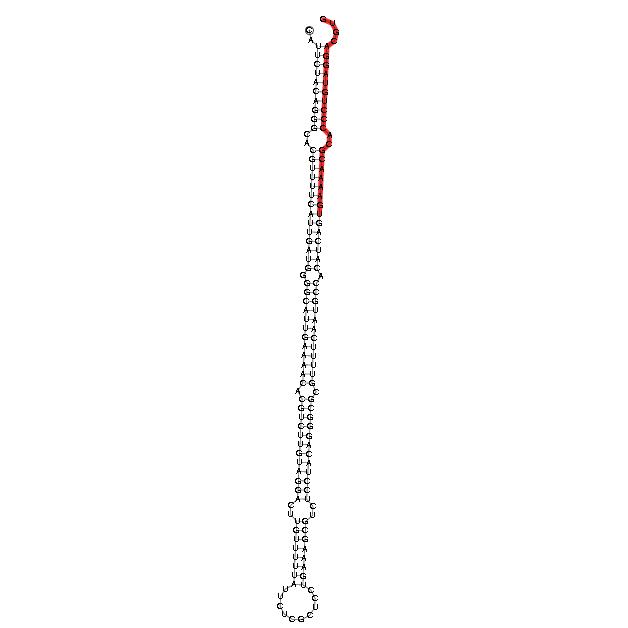

Supplement: Supplementary file 4 — Structures of all the identified novel miRNAs. (ZIP 1005 kb) [file 12864_2019_5760_MOESM4_ESM.zip › Additional file 4/ghr-miR60.jpg]

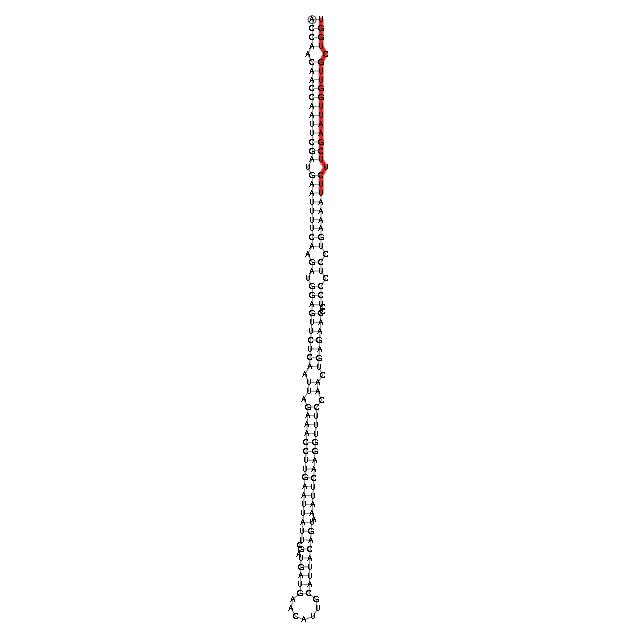

Supplement: Supplementary file 4 — Structures of all the identified novel miRNAs. (ZIP 1005 kb) [file 12864_2019_5760_MOESM4_ESM.zip › Additional file 4/ghr-miR61.jpg]

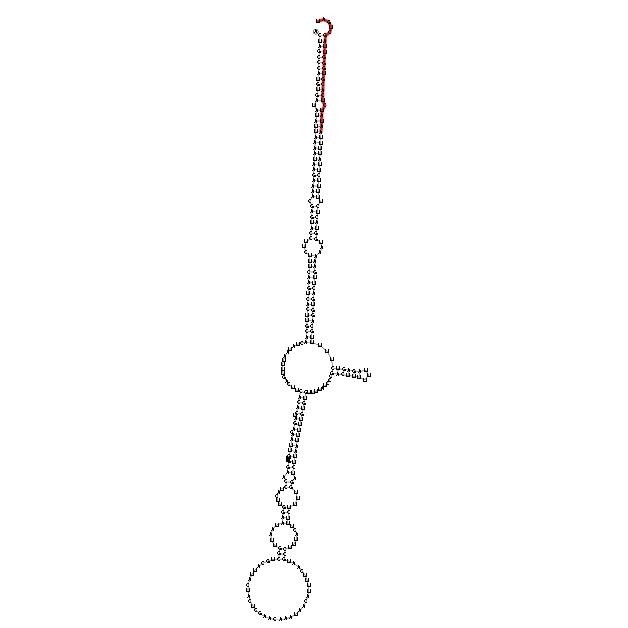

Supplement: Supplementary file 4 — Structures of all the identified novel miRNAs. (ZIP 1005 kb) [file 12864_2019_5760_MOESM4_ESM.zip › Additional file 4/ghr-miR62.jpg]

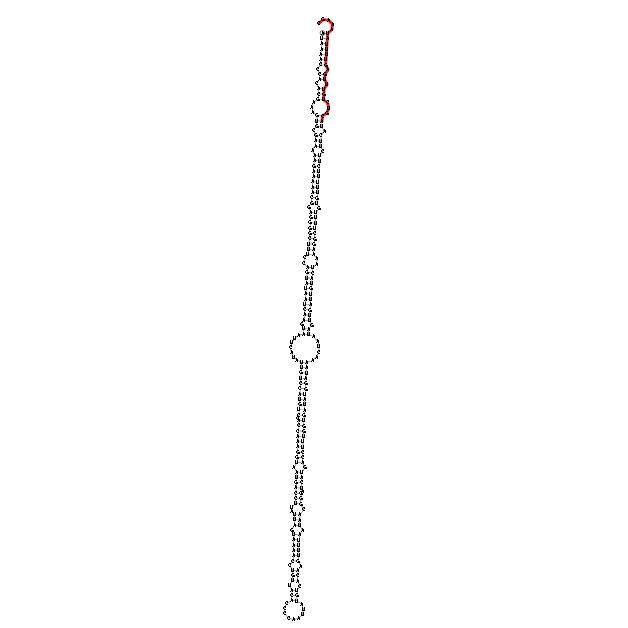

Supplement: Supplementary file 4 — Structures of all the identified novel miRNAs. (ZIP 1005 kb) [file 12864_2019_5760_MOESM4_ESM.zip › Additional file 4/ghr-miR63.jpg]

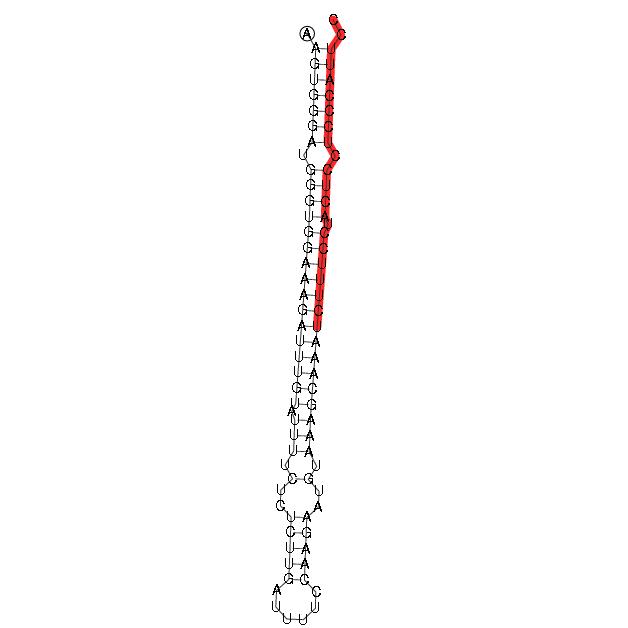

Supplement: Supplementary file 4 — Structures of all the identified novel miRNAs. (ZIP 1005 kb) [file 12864_2019_5760_MOESM4_ESM.zip › Additional file 4/ghr-miR64.jpg]

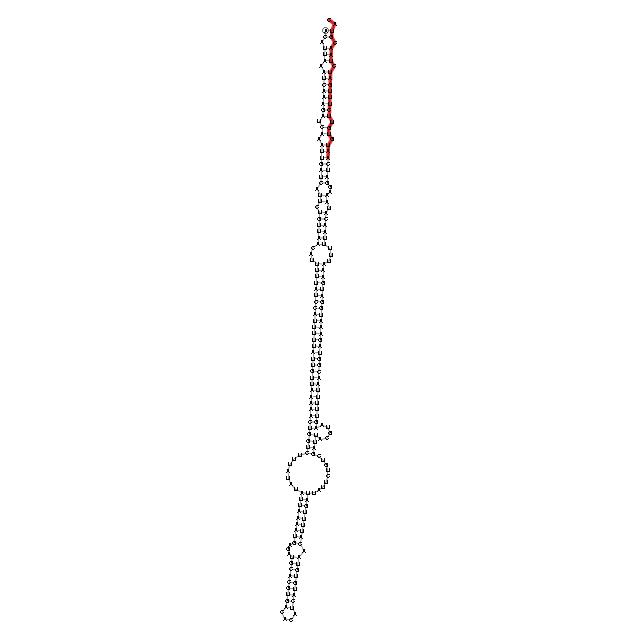

Supplement: Supplementary file 4 — Structures of all the identified novel miRNAs. (ZIP 1005 kb) [file 12864_2019_5760_MOESM4_ESM.zip › Additional file 4/ghr-miR65.jpg]

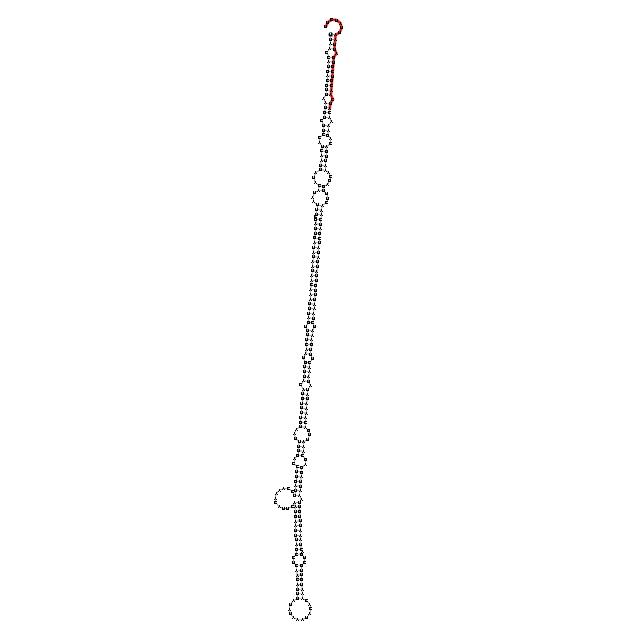

Supplement: Supplementary file 4 — Structures of all the identified novel miRNAs. (ZIP 1005 kb) [file 12864_2019_5760_MOESM4_ESM.zip › Additional file 4/ghr-miR66.jpg]

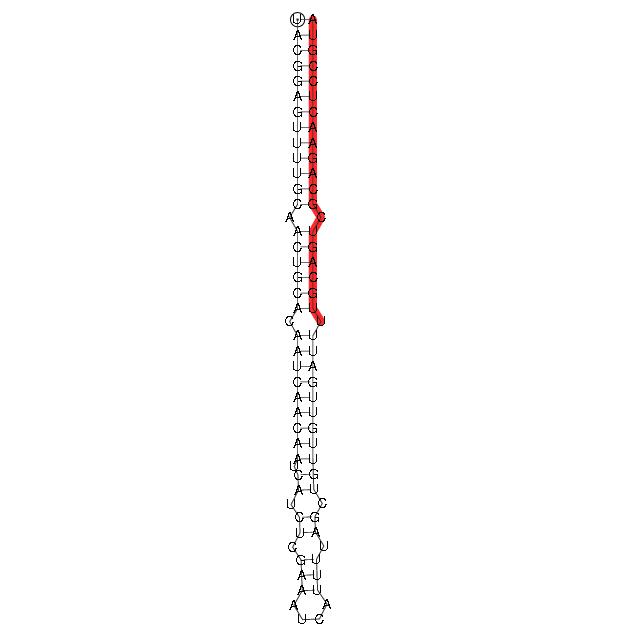

Supplement: Supplementary file 4 — Structures of all the identified novel miRNAs. (ZIP 1005 kb) [file 12864_2019_5760_MOESM4_ESM.zip › Additional file 4/ghr-miR67.jpg]

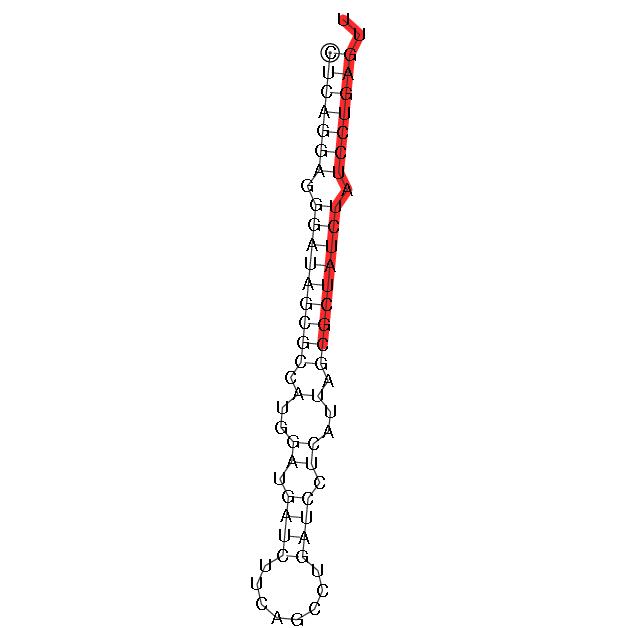

Supplement: Supplementary file 4 — Structures of all the identified novel miRNAs. (ZIP 1005 kb) [file 12864_2019_5760_MOESM4_ESM.zip › Additional file 4/ghr-miR68.jpg]

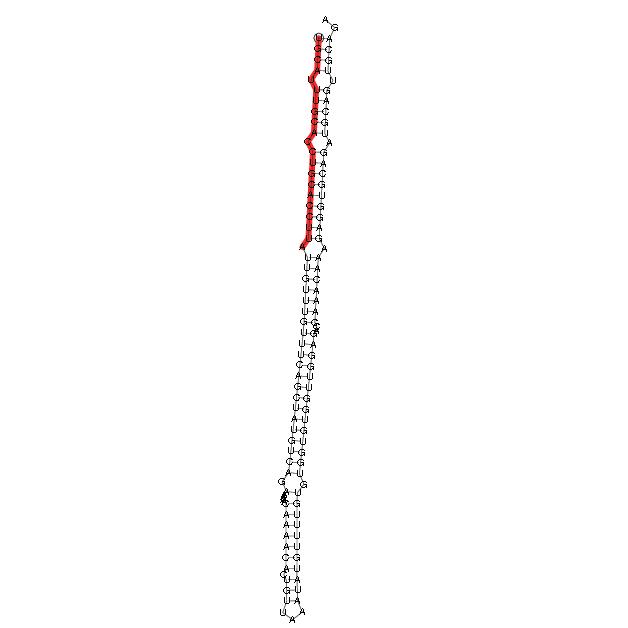

Supplement: Supplementary file 4 — Structures of all the identified novel miRNAs. (ZIP 1005 kb) [file 12864_2019_5760_MOESM4_ESM.zip › Additional file 4/ghr-miR69.jpg]

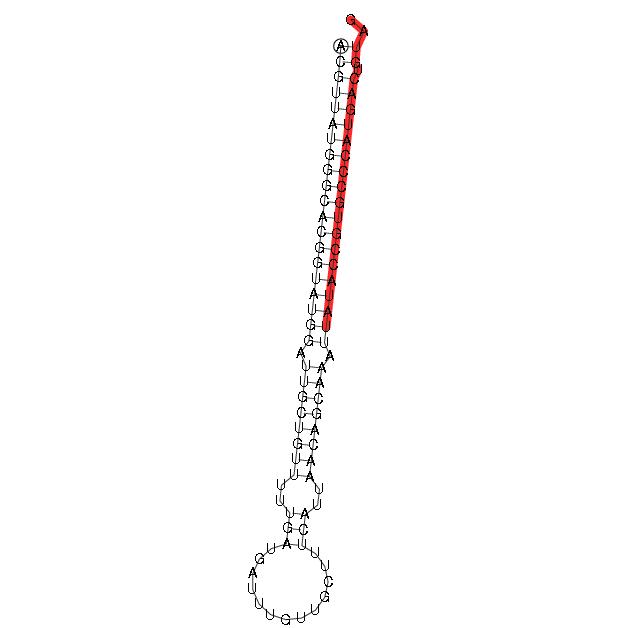

Supplement: Supplementary file 4 — Structures of all the identified novel miRNAs. (ZIP 1005 kb) [file 12864_2019_5760_MOESM4_ESM.zip › Additional file 4/ghr-miR7.jpg]

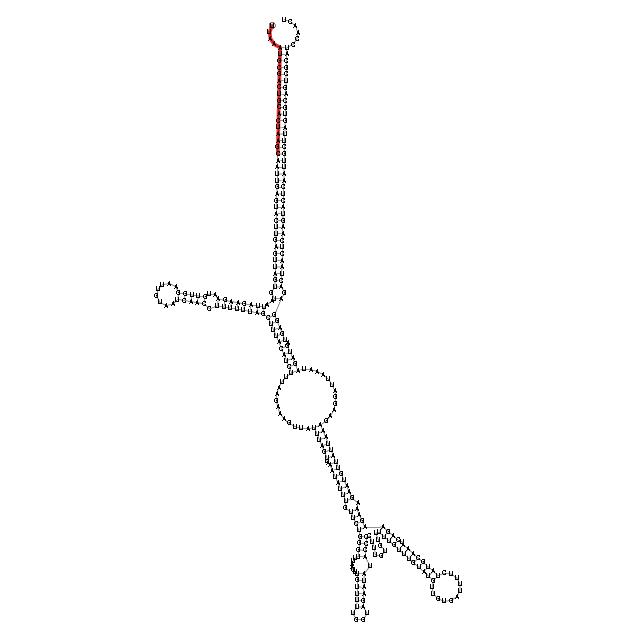

Supplement: Supplementary file 4 — Structures of all the identified novel miRNAs. (ZIP 1005 kb) [file 12864_2019_5760_MOESM4_ESM.zip › Additional file 4/ghr-miR70.jpg]

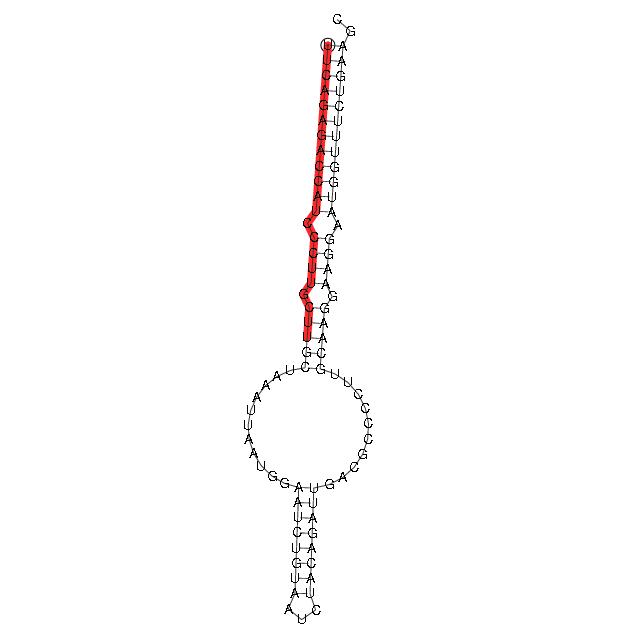

Supplement: Supplementary file 4 — Structures of all the identified novel miRNAs. (ZIP 1005 kb) [file 12864_2019_5760_MOESM4_ESM.zip › Additional file 4/ghr-miR71.jpg]

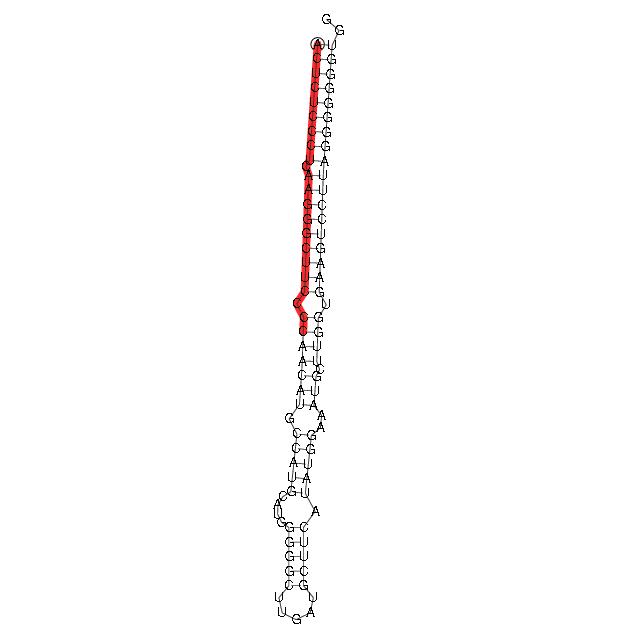

Supplement: Supplementary file 4 — Structures of all the identified novel miRNAs. (ZIP 1005 kb) [file 12864_2019_5760_MOESM4_ESM.zip › Additional file 4/ghr-miR72.jpg]

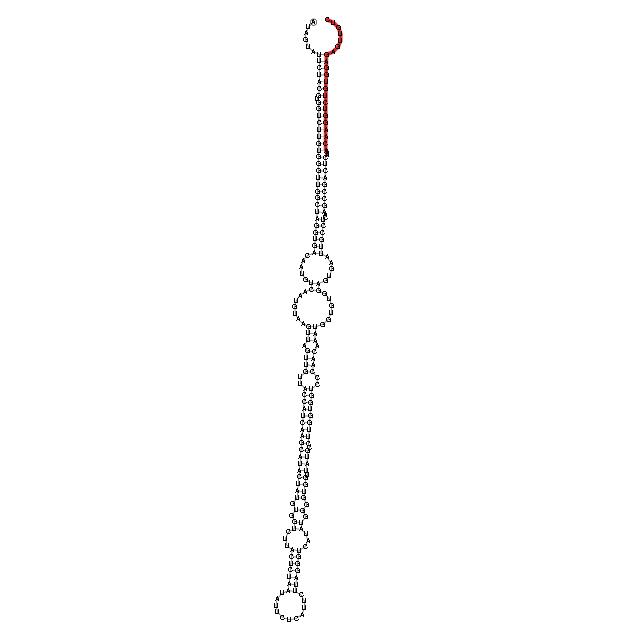

Supplement: Supplementary file 4 — Structures of all the identified novel miRNAs. (ZIP 1005 kb) [file 12864_2019_5760_MOESM4_ESM.zip › Additional file 4/ghr-miR73.jpg]

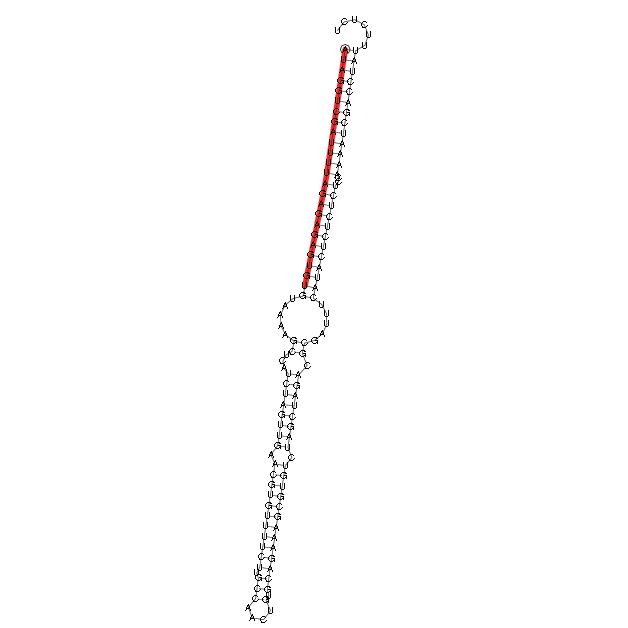

Supplement: Supplementary file 4 — Structures of all the identified novel miRNAs. (ZIP 1005 kb) [file 12864_2019_5760_MOESM4_ESM.zip › Additional file 4/ghr-miR74.jpg]

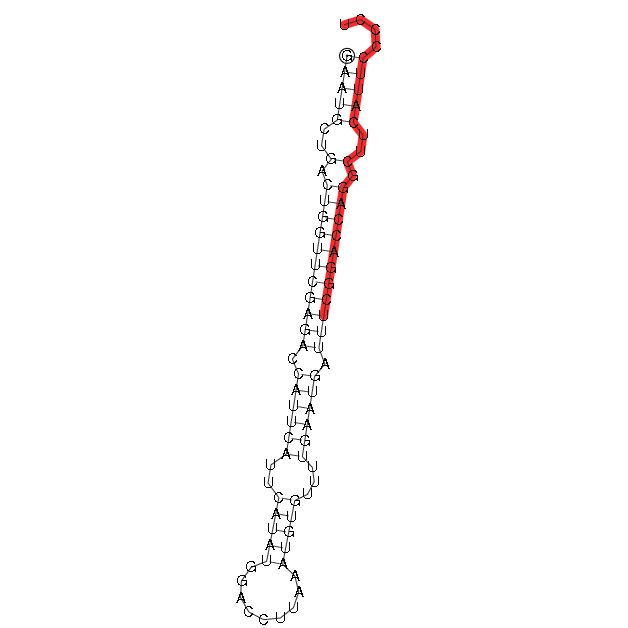

Supplement: Supplementary file 4 — Structures of all the identified novel miRNAs. (ZIP 1005 kb) [file 12864_2019_5760_MOESM4_ESM.zip › Additional file 4/ghr-miR75.jpg]

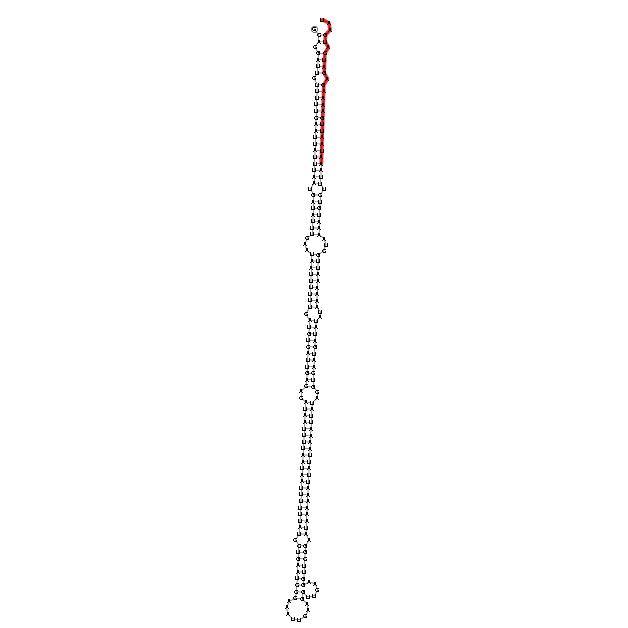

Supplement: Supplementary file 4 — Structures of all the identified novel miRNAs. (ZIP 1005 kb) [file 12864_2019_5760_MOESM4_ESM.zip › Additional file 4/ghr-miR76.jpg]

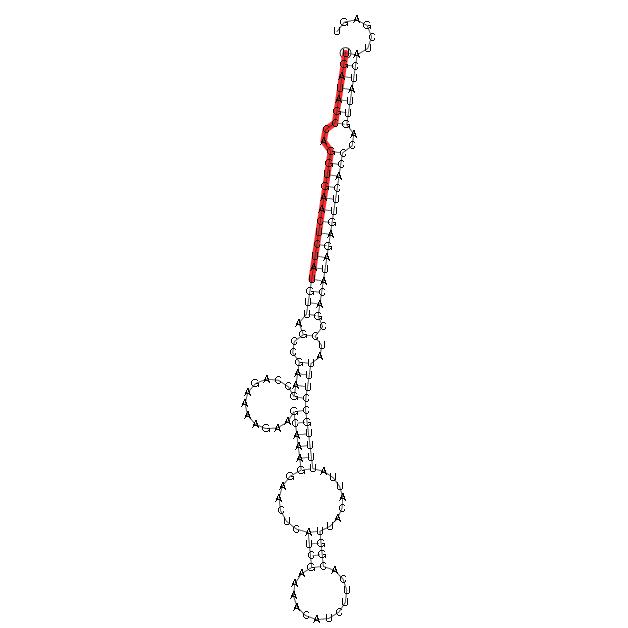

Supplement: Supplementary file 4 — Structures of all the identified novel miRNAs. (ZIP 1005 kb) [file 12864_2019_5760_MOESM4_ESM.zip › Additional file 4/ghr-miR77.jpg]

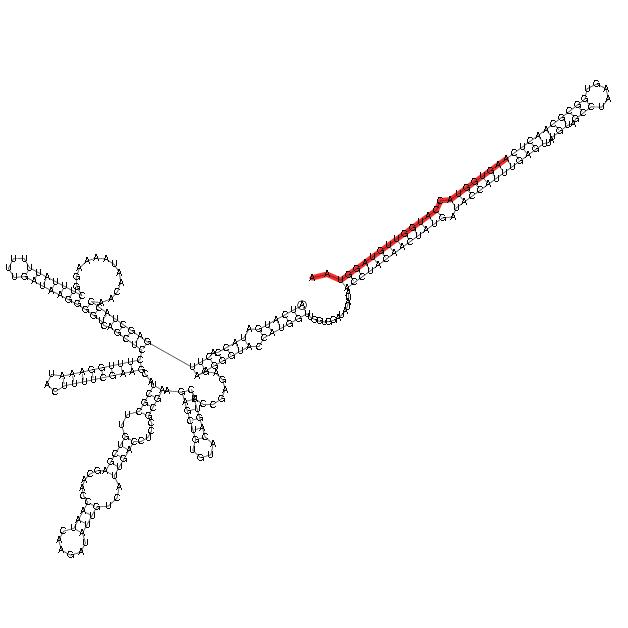

Supplement: Supplementary file 4 — Structures of all the identified novel miRNAs. (ZIP 1005 kb) [file 12864_2019_5760_MOESM4_ESM.zip › Additional file 4/ghr-miR78.jpg]

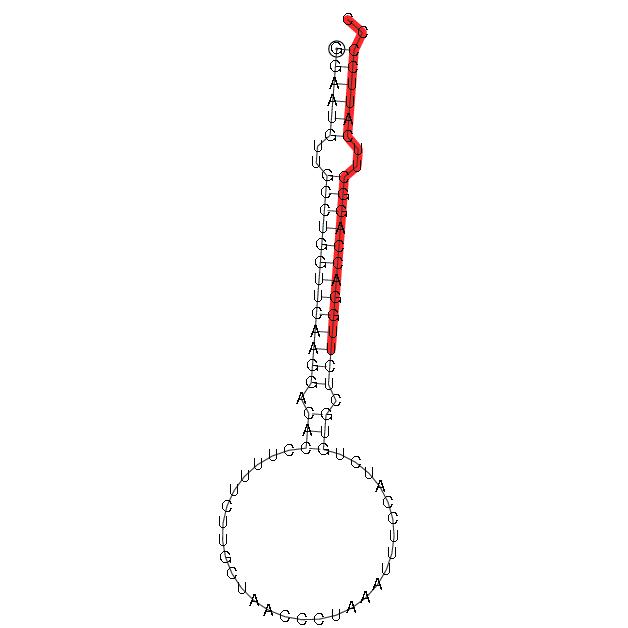

Supplement: Supplementary file 4 — Structures of all the identified novel miRNAs. (ZIP 1005 kb) [file 12864_2019_5760_MOESM4_ESM.zip › Additional file 4/ghr-miR79.jpg]

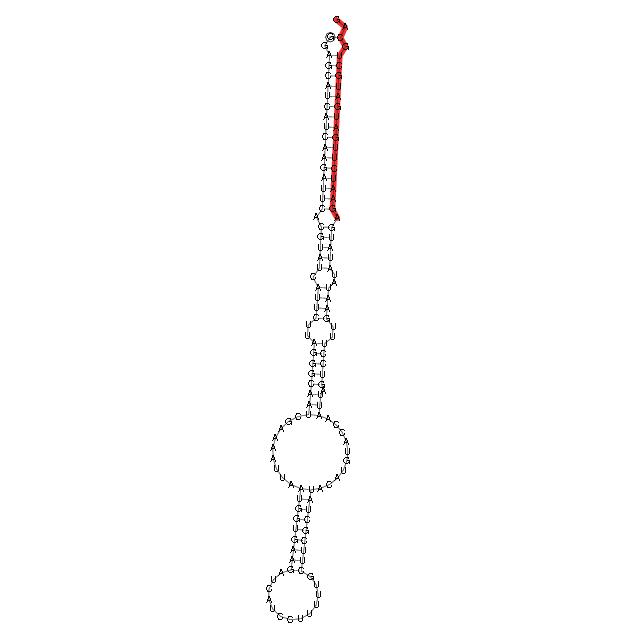

Supplement: Supplementary file 4 — Structures of all the identified novel miRNAs. (ZIP 1005 kb) [file 12864_2019_5760_MOESM4_ESM.zip › Additional file 4/ghr-miR8.jpg]

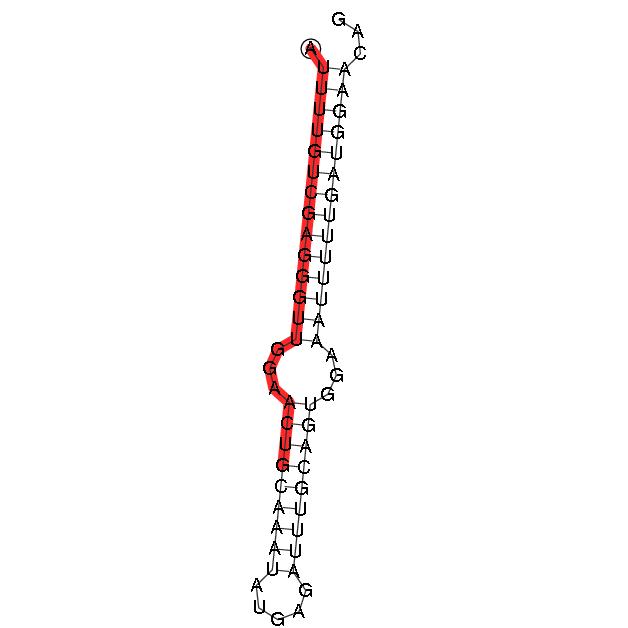

Supplement: Supplementary file 4 — Structures of all the identified novel miRNAs. (ZIP 1005 kb) [file 12864_2019_5760_MOESM4_ESM.zip › Additional file 4/ghr-miR80.jpg]

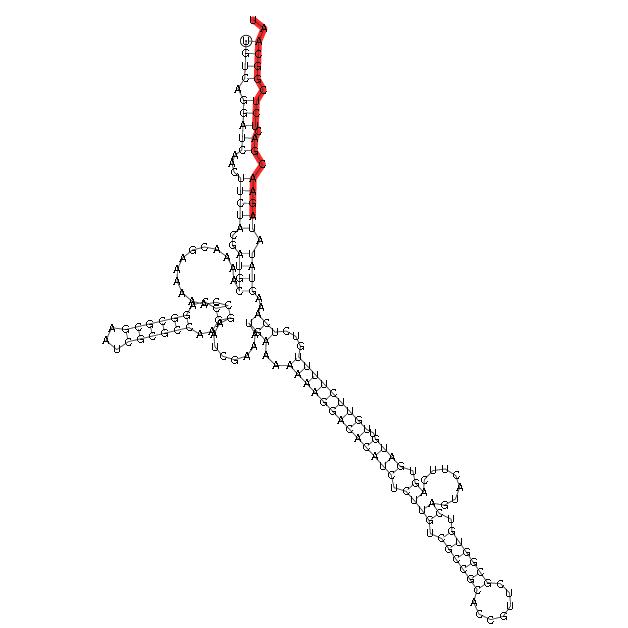

Supplement: Supplementary file 4 — Structures of all the identified novel miRNAs. (ZIP 1005 kb) [file 12864_2019_5760_MOESM4_ESM.zip › Additional file 4/ghr-miR81.jpg]

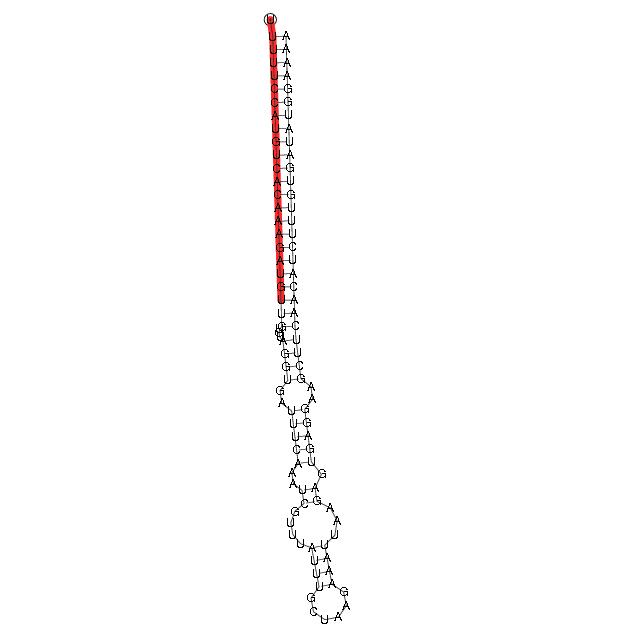

Supplement: Supplementary file 4 — Structures of all the identified novel miRNAs. (ZIP 1005 kb) [file 12864_2019_5760_MOESM4_ESM.zip › Additional file 4/ghr-miR82.jpg]

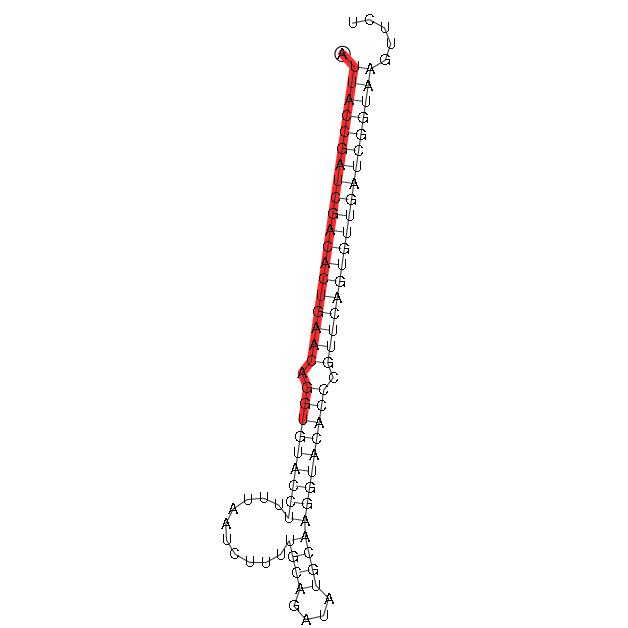

Supplement: Supplementary file 4 — Structures of all the identified novel miRNAs. (ZIP 1005 kb) [file 12864_2019_5760_MOESM4_ESM.zip › Additional file 4/ghr-miR83.jpg]

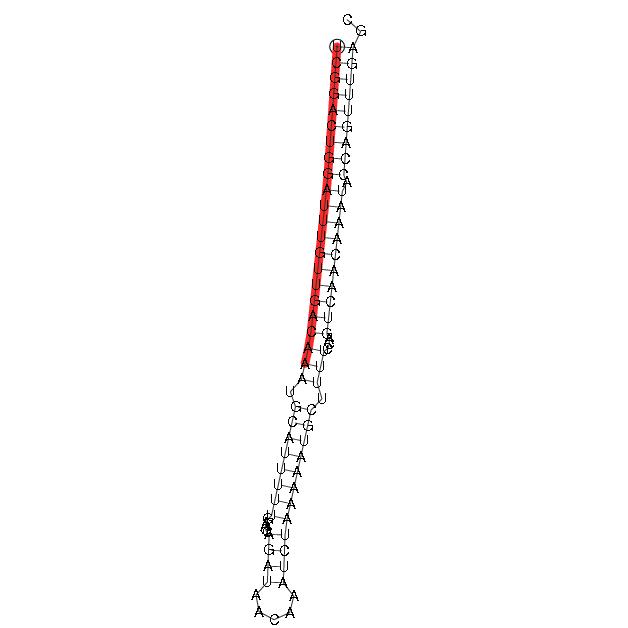

Supplement: Supplementary file 4 — Structures of all the identified novel miRNAs. (ZIP 1005 kb) [file 12864_2019_5760_MOESM4_ESM.zip › Additional file 4/ghr-miR9.jpg]
